# Reversible C–CN Bond Cleavage by a Formal Dinickel(I) Hydride Cation

**DOI:** 10.1021/acs.organomet.4c00340

**Published:** 2024-11-01

**Authors:** Yu Cao, Neil A. Dodd, John Bacsa, Joseph P. Sadighi

**Affiliations:** † School of Chemistry and Biochemistry, Georgia Institute of Technology, Atlanta, Georgia 30332-0400, United States; ‡ X-ray Crystallography Center, Department of Chemistry, Emory University, 1515 Dickey Drive, Atlanta, Georgia 30322, United States

## Abstract

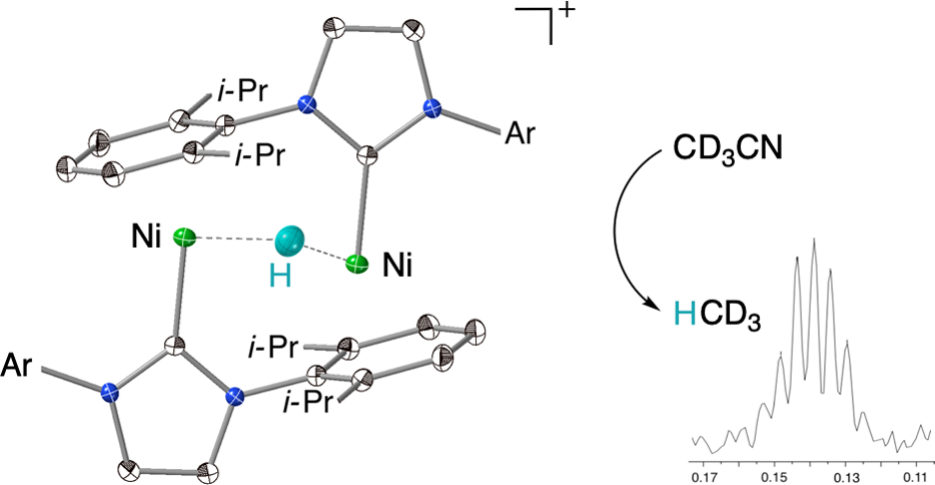

An N-heterocyclic carbene (NHC) ligand supports a stable [Ni_2_H]^+^ core, formally dinickel(I). This diamagnetic cation complex features a bent hydride bridge and a Ni···Ni distance, 2.9926(5) Å, larger than two covalent radii. The cation displays weakly protic character, undergoing deprotonation by strong base to form the corresponding (NHC)nickel(0) dimer. Its reaction with aliphatic nitriles results in C–CN bond cleavage. The organic products of this reaction suggest that this bond-breaking step involves reactive nickel alkyl intermediates and occurs reversibly.

## Introduction

The oxidative addition of nitrile C–CN bonds to nickel(0) represents a notable class of metal-mediated carbon–carbon bond activation, with importance to organic synthesis.^1^ The reverse of this process, the reductive elimination of a C–CN bond, is crucial to the hydrocyanation of alkenes,^2^ alkynes,^3^ and allenes.^4^ Catalyst systems that readily accomplish both the forward and reverse reactions enable carbocyanation^5^ and transfer hydrocyanation.^6^ In both processes, a nitrile serves as a benign source of cyanide in the construction of more valuable nitriles.

Nickel hydrides^7^ are key intermediates in hydrocyanation, and in a range of hydrogenation^8^ and hydrofunctionalization^9^ catalysis. They can also serve as sources of lower-valent nickel through loss of hydrogen.^10^ In pioneering studies of nitrile activation by Garcia and Jones,^11^ a (bisphosphine)nickel(I) hydride dimer reductively eliminates H_2_ to form nickel(0) fragments that readily undergo oxidative addition.^12^ An unusual nickel(III) hydride, described by Peters and co-workers, can generate H_2_ either through protonolysis or through bimolecular reductive elimination.^13^ As intermediates in proton reduction,^14^ nickel hydrides are relevant to the action of Ni/Fe hydrogenases,^15^ and have been detected in the active site of methyl-coenzyme M reductase.^16^

While exploring the chemistry of low-valent nickel complexes, we examined the ability of N-heterocyclic carbenes (NHCs) to support reactive nickel hydrides. Nickel complexes were among the early coordination compounds prepared from isolable NHCs.^17^ The (NHC)Ni platform features prominently in catalytic reactions of alkenes^18^ and alkynes,^19^ and in difficult oxidative addition reactions. The cleavage of carbon–fluorine bonds^20^ is key to several fluorocarbon transformations; that of carbon–oxygen bonds^21^ is relevant to the conversion of biomass to fuels.

We became interested in the potential reactivity of nickel(I).^22^ An early attempt to prepare an (NHC)nickel(I) hydride dimer resulted instead in the reductive elimination of H_2_, leaving an (NHC)nickel(0) dimer.^23^ We wondered whether a hydride-bridged dinickel(I) cation would be isolable. The hydride-bridged dinickel(0) anions {(μ-H)[Ni(η^2^-C_2_H_4_)_2_]_2_}^−^^24^ and {(μ-H)[Ni(CO)_3_]_2_}^−^^25^ are well characterized, and hydride-bridged dicopper(I)^26^ and disilver(I)^27^ cations had proven more thermally robust than their neutral analogues. The loss of H_2_ from a [Ni_2_(μ-H)]^+^ complex would require either the intermolecular approach of two sterically encumbered cations to form an H–H bond, or the activation of a ligand C–H bond. We expected the kinetic barrier for either pathway to be high.

We now report the synthesis of a hydride-bridged dinickel cation, in which the N-aryl substituents of the NHC ligands provide a symmetrically bridging framework. This formally dinickel(I) complex is diamagnetic, consistent with three-center, four-electron bonding among the nickel and hydrogen atoms. Its reaction with nitriles leads to C–C bond cleavage, with the release of alkane and other products suggestive of a reactive nickel alkyl intermediate.

## Results and Discussion

**Scheme 1:**
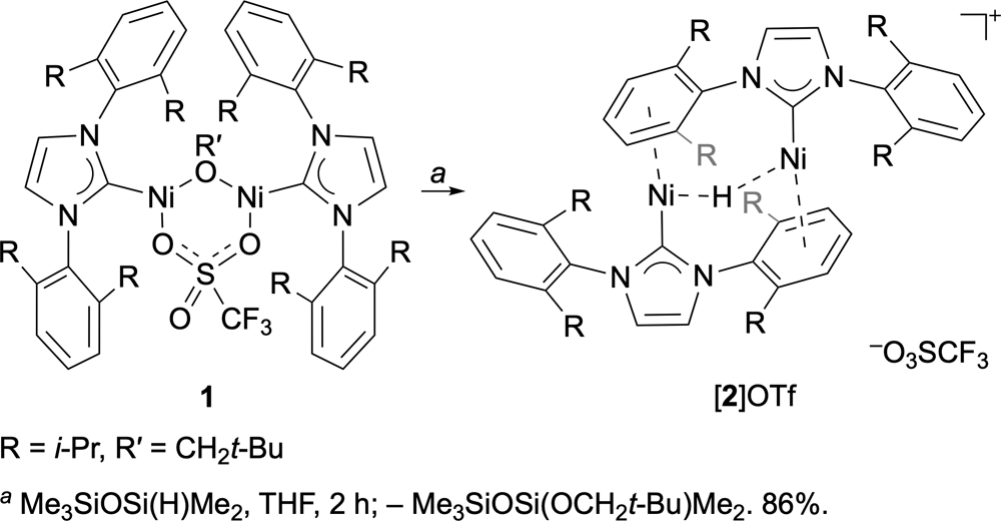
Synthesis of a Hydride-Bridged Dinickel Cation

The alkoxy/triflate-bridged dinickel(I) complex {[(IDipp)Ni]_2_(μ-OCH_2_*t*-Bu)(μ-O_3_SCF_3_)}^28^ (**1**, IDipp = 1,3-bis(2,6-diisopropylphenyl)imidazol-2-ylidene^29^) reacts with silane and borane derivatives, such as phenylsilane and pinacolborane, to exchange alkoxide for hydride. Pentamethyldisiloxane in particular gave smooth conversion (Scheme 1), and allowed facile isolation of the hydride complex [**2**]OTf. The ^1^H NMR spectrum of [**2**]OTf (Figure S1, Supporting Information) displays sharp peaks, including well-defined multiplets, consistent with a diamagnetic species. A singlet resonance at δ −25.6 ppm, integrating to one proton per two IDipp ligands, arises from the bridging hydride.

Some features in the ^1^H NMR spectrum were initially puzzling. The ligand methyl groups give rise to three doublet resonances, in an intensity ratio of 1:1:2, between δ 1.5 and 1.1 ppm. Restricted rotation about the C–N bonds of IDipp typically gives rise to two distinct doublets: Four methyl groups point toward the bound metal, four point away, and these do not exchange positions on the NMR time scale. Restrictions in rotation about the metal–carbon bonds might cause four distinct methyl resonances to appear. The presence of three doublets suggested some less straightforward symmetry breaking. The origin of a triplet resonance (2H, *J* = 7.2 Hz) at δ 4.80 ppm was not immediately clear: Normally the alkyl hydrogen resonances would appear well upfield of this chemical shift, the aryl and NHC backbone hydrogens well downfield.

**Figure 1:**
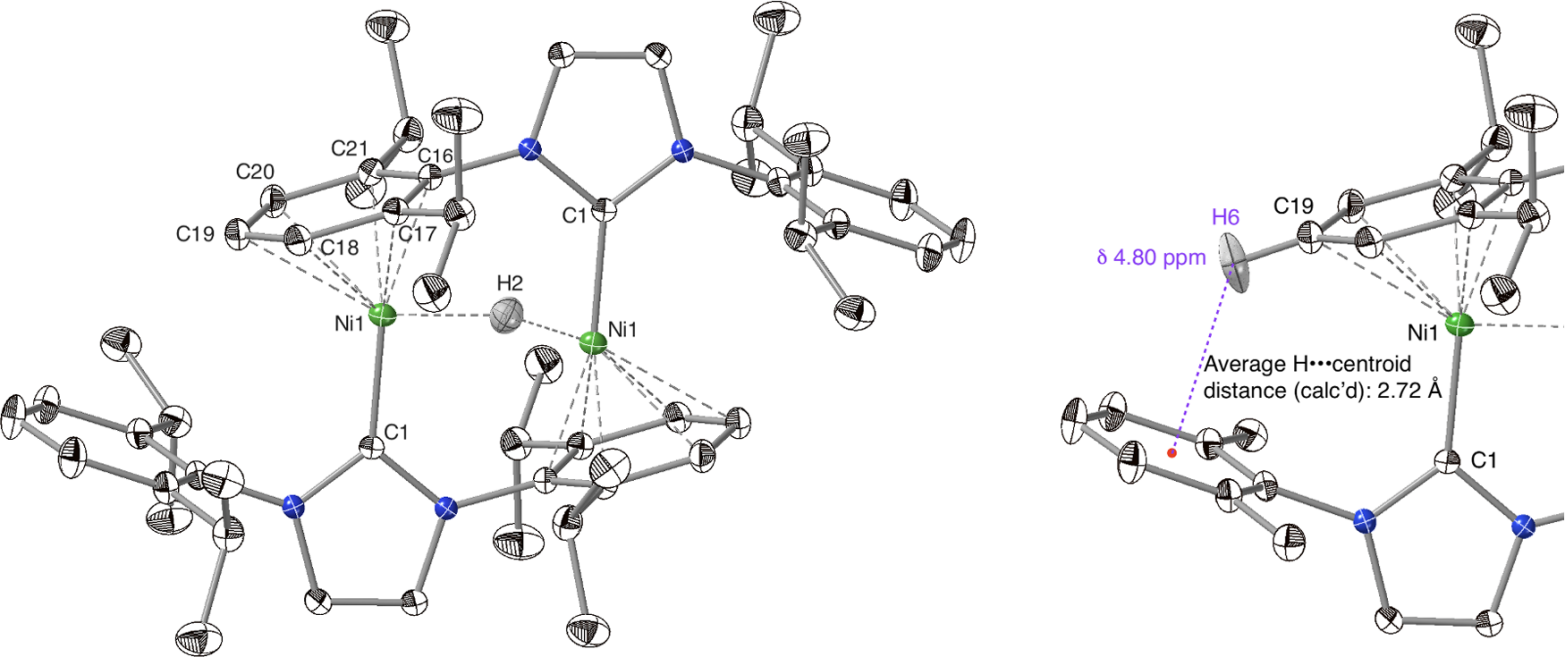
Solid-state structure of {[(IDipp)Ni]_2_(μ-H)}^+^ (**2**), shown as 50% probability ellipsoids. Triflate anion omitted for clarity. Selected interatomic distances (Å) and angles (°): Ni1–H2 1.34(2), 1.75(2), Ni1–C1 1.8928(10), Ni1–C16 2.0554(10), Ni1–C19 2.3680(11), Ni1–Ni1 2.9926(5); Ni1–H2–Ni1 152(2), C1–Ni1–Ni1 85.15(3).

The solid-state structure of [**2**]OTf (Figure 1) reveals an arrangement of rough *C*_2h_ symmetry, in which each NHC ligand serves as carbene donor to one nickel center while offering an *N*-aryl group as a ligand to the other. The same arrangement is found in the nickel(0) dimer [(IDipp)Ni]_2_,^23^ and in its one-electron oxidation product, {[(IDipp)Ni]_2_}^+^.^30^ The intermetallic distance of 2.9926(5) Å is smaller than two van der Waals radii for Ni (3.26 Å),^31^ but well outside two covalent radii (2.48 Å).^32^ This Ni···Ni distance falls between those of [(IDipp)Ni]_2_ (no Ni–Ni bond: 3.568(2) Å) and {[(IDipp)Ni]_2_}^+^ (Ni–Ni half-bond: 2.6359(5) Å). The Ni–C_NHC_ distance in **2** is 1.893(1) Å, and the Ni–C_N–Ar_ distances range from 2.055(1) to 2.368(1) Å.

The C_NHC_–Ni–Ni angle is acute at 85.14(3)°. The angle formed by the carbene carbon, nickel, and the calculated centroid of the bound *N*-aryl ring is 152.1°. The triflate counterion, disordered, lies outside the nickel coordination spheres.

This structure suggests an explanation for key features of the solution ^1^H NMR spectrum. The eight methyl groups pointing away from the [Ni_2_H] core comprise two sets of four, but their chemical shifts could easily coincide. Of the eight methyl groups pointing toward the core, the four that flank the hydride are in a clearly different chemical environment from those pointing away. This explanation is consistent with the observation of three doublet resonances in a ratio of 24:12:12. The triplet resonance at δ 4.80 ppm is assigned to the *para*-proton of each Ni-bound *N*-aryl group. The calculated position (C19–H; see Figure 1) lies only 2.72 Å from the centroid of an unbound *N*-aryl ring. If the geometry of **2** were the same in solution as in the solid state, these two aryl protons would each be locked into the diatropic shielding region of an aromatic ring, consistent with their strong upfield shift.^33^ In the solid-state structure of [(IDipp)Ni]_2_ the corresponding distance is longer, 3.03 Å, and the triplet resonance for these *N*-aryl protons is shifted less dramatically, to δ 5.22 ppm.^23^

The bridging hydride is disordered over two equivalent positions. These positions refine to give unequal distances to the nickel centers, 1.34(2) and 1.75(2) Å. The Ni–H–Ni angle of 150(2)° is notably larger than the Cu–H–Cu angle of 122(3)° in {[(IDipp)Cu]_2_(μ-H)}^+^.^26b^ In many dinuclear complexes with single hydride bridges,^34^ a bent [M(μ-H)M] core gives rise to stronger bonding. Vicic’s mixed-valence Ni(I)/Ni(II) μ-hydride complexes represent rare exceptions, featuring linear hydride bridges.^35^ If the [Ni(μ -H)Ni]^+^ core of **2** represents a half-filled orbital from each d^9^ nickel center, and a filled hydrogen 1s orbital, the resulting three-center, four-electron species has no net metal–metal bond and no obvious reason to prefer a bent geometry.^36^ The bridging IDipp ligands could accommodate a larger Ni···Ni distance, as they do in [(IDipp)Ni]_2_. A more nearly linear arrangement is plausible for **2**, but apparently not favored.

The bridging hydrogen in **2** does not exhibit hydridic character in its reactivity. Addition of CD_3_OD to a THF-*d*_8_ solution of [**2**]OTf, in a J. Young NMR tube sealed immediately afterward, gave rise to no discernible signal for H–D in the resulting ^1^H NMR spectrum. Attempted reaction of [**2**]OTf with 1.1 atm CO_2_ showed largely unreacted starting material, with partial decomposition to a complex mixture after several hours at 60 °C. For comparison, the addition of CD_3_OD to {[(IDipp)Cu]_2_(μ-H)}^+^ leads to the appearance of a 1:1:1 triplet resonance^37^ for H–D in the ^1^H NMR spectrum, and exposure of the same cation to CO_2_ affords a formate-bridged dicopper cation.^26b^

The nickel-bound hydrogen in **2** may also be viewed as a proton shared between two basic Ni(0) centers, an arrangement in which two filled nickel d-orbitals interact with the vacant hydrogen 1s orbital.^38^ Meyer and co-workers recently described the protonation of a nickel(0) center bearing a tripodal NHC ligand to form a nickel(II) hydride, and subsequently a dihydrogen complex.^39^ Protonation of [(IDipp)Ni]_2_, or [(IDipp)Ni(η^6^-C_6_H_6_)], in benzene solution by ethereal HNTf_2_ affords [**2**]NTf_2_. This reaction also affords varying fractions of {[(IDipp)Ni]_2_}^+^^30^ as a byproduct, and the similar size and shape of this cation to **2** make it difficult to separate salts of the two. We have therefore focused on [**2**]OTf for reactivity studies. Nonetheless, in all preparations of [**2**]NTf_2_ the distinctive singlet resonance at δ −25.3 ppm in the ^1^H NMR spectrum, and well-defined IDipp resonances (Figure S5), confirmed the generation of **2** from two nickel(0) centers plus a proton donor. Conversely, deprotonation of **2** regenerates two nickel(0) centers: Treatment of [**2**]OTf with NaN(SiMe_3_)_2_ in THF solution forms [(IDipp)Ni]_2_^23^ as the sole IDipp-containing species identified by ^1^H NMR spectroscopy (Figure S8). Thus, the nickel(0) dimer and a cationic dinickel(I) hydride can be interconverted through proton transfer.

Exposure of [**2**]OTf in THF-*d*_8_ solution to an atmosphere of carbon monoxide resulted in the breakup of the complex (eq 1) after 10 min. Reductive elimination of the hydride and one IDipp ligand forms the imidazolium salt [(IDipp)H]^+^ OTf^–^. Here again, the hydride exhibits formal protic character, converting an NHC ligand to its conjugate acid. The resulting Ni(CO)_4_ was identified by ^13^C NMR,^40^ [(IDipp)Ni(CO)_3_]^17^ by ^1^H and ^13^C NMR spectroscopy (Figures S9 and S10).
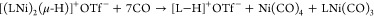
1

The reaction of **2**with aliphatic nitriles results in cleavage of the C–CN bond. Garcia, Jones and co-workers have demonstrated the oxidative addition of C–CN bonds to (bisphosphine)nickel(0) centers,^41^ including several generated by reductive elimination of H_2_ from nickel(I) hydride dimers. Louie and co-workers have shown that oxidative addition is not the preferred pathway for the interaction of a nitrile with (IDipp)Ni(0). Instead, the nickel(0) binds the C≡N fragment side-on, and the resulting η^2^-nitrile complex is activated toward cycloaddition reactions.^42^ In contrast In contrast, Garcia and co-workers did observe oxidative addition of an allylic C–CN bond to (NHC)nickel complexes, forming a nickel(II) (allyl) cyanide.^43^

**Figure 2:**
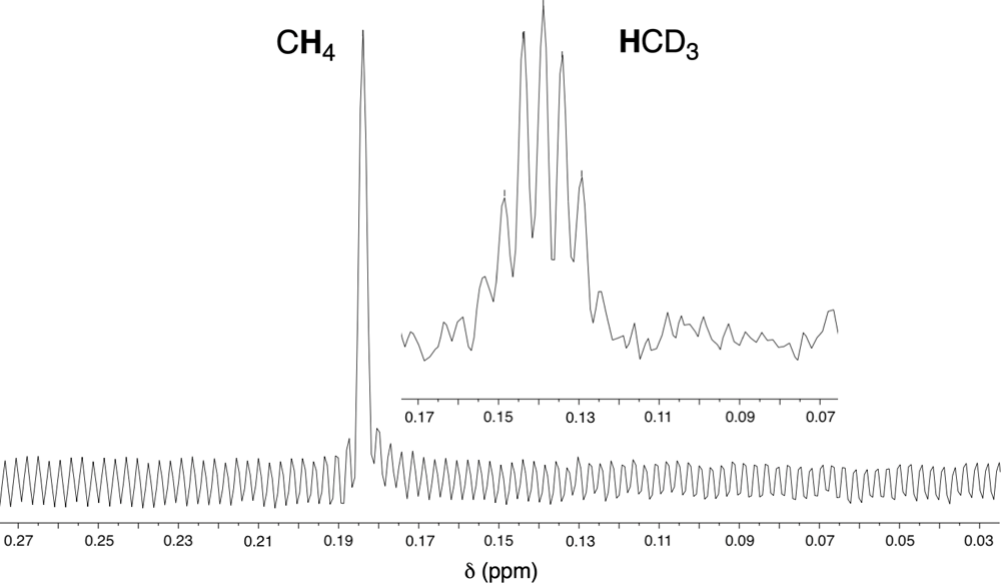
^1^H NMR resonances for CH_4_, formed from CH_3_CN, and HCD_3_, formed from CD_3_CN. Taken from spectra obtained in THF-*d*_8_ solution (Figures S11 and S12, Supporting Information).

In the case of **2**, C–CN bond cleavage leads to alkane formation. Thus, the reaction of acetonitrile with [**2**]OTf in THF-*d*_8_ solution formed methane, with a singlet ^1^H NMR resonance at δ 0.18 ppm (Figure S11), plus a complex mixture of (IDipp)Ni-derived decomposition products. To confirm the origin of the methyl group, we carried out the reaction with CD_3_CN and observed the septet resonance for CD_3_H^44^ at δ 0.14 ppm (Figure 2;^2^*J*_H–D_ = 2.1 Hz).

**Scheme 2:**
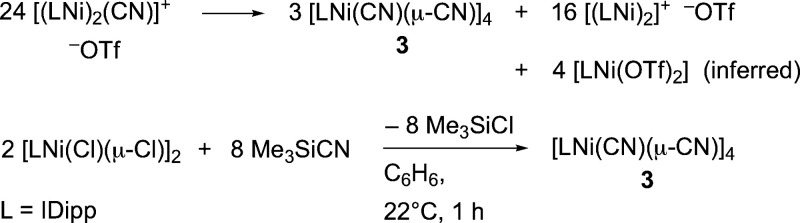
In Situ and Deliberate Synthesis of [LNi(CN)_2_]_4_

In principle, the release of methane from CH_3_CN plus {[(IDipp)Ni]_2_(μ-H)}^+^ should leave a cyanide-bridged dinickel(I) cation. We have not observed such a species; if formed, it disproportionates rapidly (Scheme 2). From reaction mixtures with longer-chain nitriles (see below), we have crystallized the mixed-valent dinickel(I,0) cation {[(IDipp)Ni]_2_}^+^, described previously.^30^ Other reaction mixtures deposited colorless crystals of a nickel(II) cyanide complex (**3**). To evaluate our hypothesis that {[(IDipp)Ni]_2_(μ-CN)}^+^ is unstable with respect to disproportionation, we attempted its synthesis independently. The reaction of **1** with Me_3_SiCN, which should form {[(IDipp)Ni]_2_(μ-CN)}^+^ plus Me_3_SiOCH_2_*t*-Bu, gave rise to ^1^H NMR spectra (Figures S16 and S17) very similar to those obtained from reaction mixtures of **2** with nitriles.

Next, we carried out the independent synthesis of the nickel cyanide **3**. The reaction of [(IDipp)Ni(Cl)(μ-Cl)]_2_ with Me_3_SiCN in benzene solution proceeded cleanly, releasing Me_3_SiCl and allowing isolation of **3**, a colorless solid, in 76% yield. Curiously, the product as initially isolated gave a well-resolved ^1^H spectrum with a sharp resonance at δ 0.31 ppm, integrating to nearly one equivalent of Me_3_Si, and small, poorly defined resonances adjacent to those assigned to the IDipp ligand (Figure S18). Recrystallization results in the disappearance of both the [Me_3_Si] resonance and the sharply defined ligand resonances, and the poorly defined resonances for the IDipp ligand predominate (Figure S20). Samples of recrystallized product pass elemental analysis for [(IDipp)Ni(CN)(μ-CN)]. We believe that the procedure initially affords an adduct [(IDipp)Ni(CN)_2_·(NCSiMe_3_)], with well-defined resonances, and that this adduct loses silyl cyanide to form the square tetramer **3**, with multiple similar but inequivalent environments for the NHC ligands.

**Figure 3:**
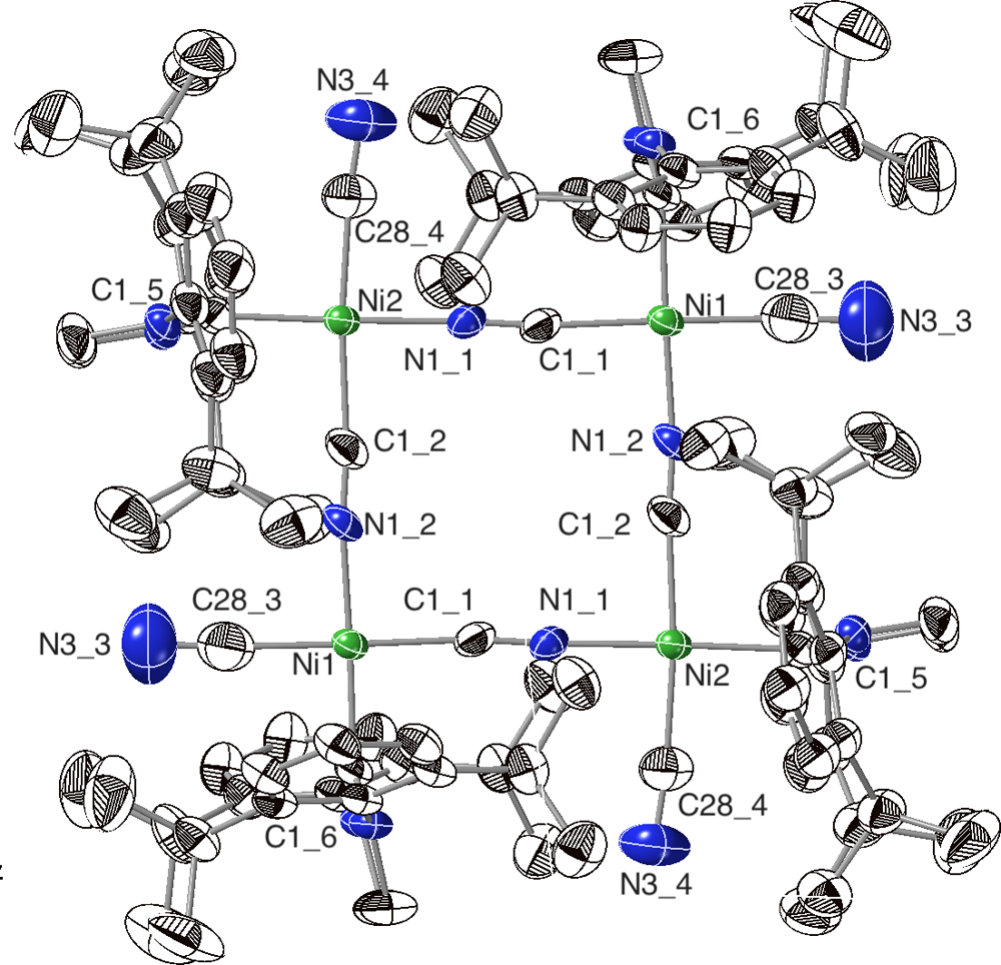
Solid-state structure of [(IDipp)Ni(CN)(μ-CN)]_4_ (**3**), shown as 50% probability ellipsoids. Selected interatomic distances (Å) and angles (°): Ni1–C28_3, 1.882(12), C28_3–N3_3, 1.160(18), Ni1–C1_6, 1.907(6), Ni1–C1_1, 1.903(8), C1_1–N1_1, 1.124(11), Ni1–N1_2, 1.841(6); C1_1–Ni1–C1_6, 90.3(3), C1_6–Ni1–C28_3, 90.8(4), C28_3–Ni1–N1_2, 87.5(4), N1_2–Ni1–C1_1, 91.4(3), Ni1–C1_1–N1_1, 174.2(6).

Crystals of **3** were prepared by layering a THF solution with pentane. These crystals were unstable, and X-ray diffraction gave low precision. The resulting solid-state structure (Figure 3) nonetheless sufficed to show the connectivity of **3**. The complex forms a cyanide-bridged tetranuclear square,^45^ in which each nickel(II) center bears an IDipp and a terminal cyanide ligand, and is connected to its neighbors by bridging cyanides. These bridges are disordered, with each atomic position representing a mixture of C and N. One structural solution, in which the terminal CN is *trans* to one bridging cyanide carbon, is shown in Figure 2; another, in which this arrangement is *cis*, is given in the Supporting Information (Figure S22).

**Figure 4:**
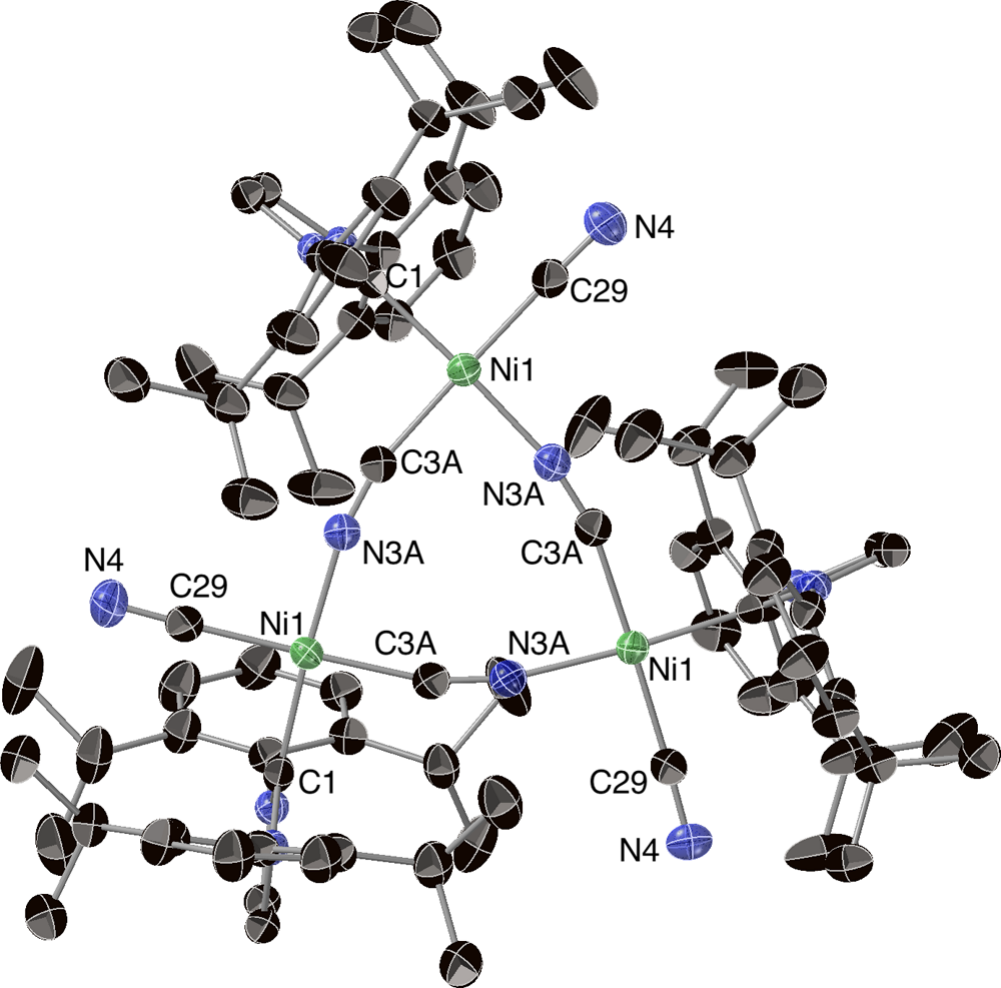
Solid-state structure of [(IDipp)Ni(CN)(μ-CN)]_3_ (**3′**), shown as 50% probability ellipsoids. Selected interatomic distances (Å) and angles (°): Ni1–C1, 1.885(3), Ni1–C3A, 1.885(2), Ni1–N3A, 1.888(1), Ni1–C29, 1.853(3), C3A–N3A, 1.134(3), C29–N4, 1.152(4); C1–Ni1–N3A, 174.69(9), C1–Ni1–C3A, 91.28(8), C3A–Ni1–N3A, 85.62(9), Ni1–C29–N4, 177.6(2), Ni1–N3A–C3A, 167.1(2), N3A–C3A–Ni1, 165.9(2).

Subsequent recrystallization of **3** by solvent vapor diffusion, to grow higher-quality crystals for a more precise structure, gave a surprising result. The solid-state structure now revealed the cyclic trimer structure **3′** (Figure 4), still composed of (IDipp)Ni(CN)(μ-CN) monomers. The nickel(II) centers in **3′** are nearly square, with an angle of 85.62(9)° between the bridging cyanides. The bonds about the bridging cyanides are bent as well, with Ni–C–N angles of 165.9(2)°, and Ni–N–C angles of 167.1(2)°. These distortions from square planar nickel and linear bridges enable the trimer to form instead of the tetramer. In the extended structure (Figure S23), unit cells containing two trimers are linked by noncovalent interactions to form interlocking rings. The formation of different oligomeric structures by the nickel(II) cyanide under very similar conditions is consistent with the complexity of its solution ^1^H NMR spectrum.

Besides the usual features resulting from the IDipp ligand, the infrared spectrum of **3** (Figure S21) displayed a sharp absorbance at 2156 cm^–1^, consistent with the stretching of terminal C≡N bonds. Weaker shoulders at 2147 and 2143 cm^–1^ may arise from the bridging cyanides. The infrared spectrum of [Ni(CN)_2_·1.5H_2_O], for comparison, displays a strong absorbance at 2165 cm^–1^ and a weak one at 2125 cm^–1^.^46^

**Scheme 3:**
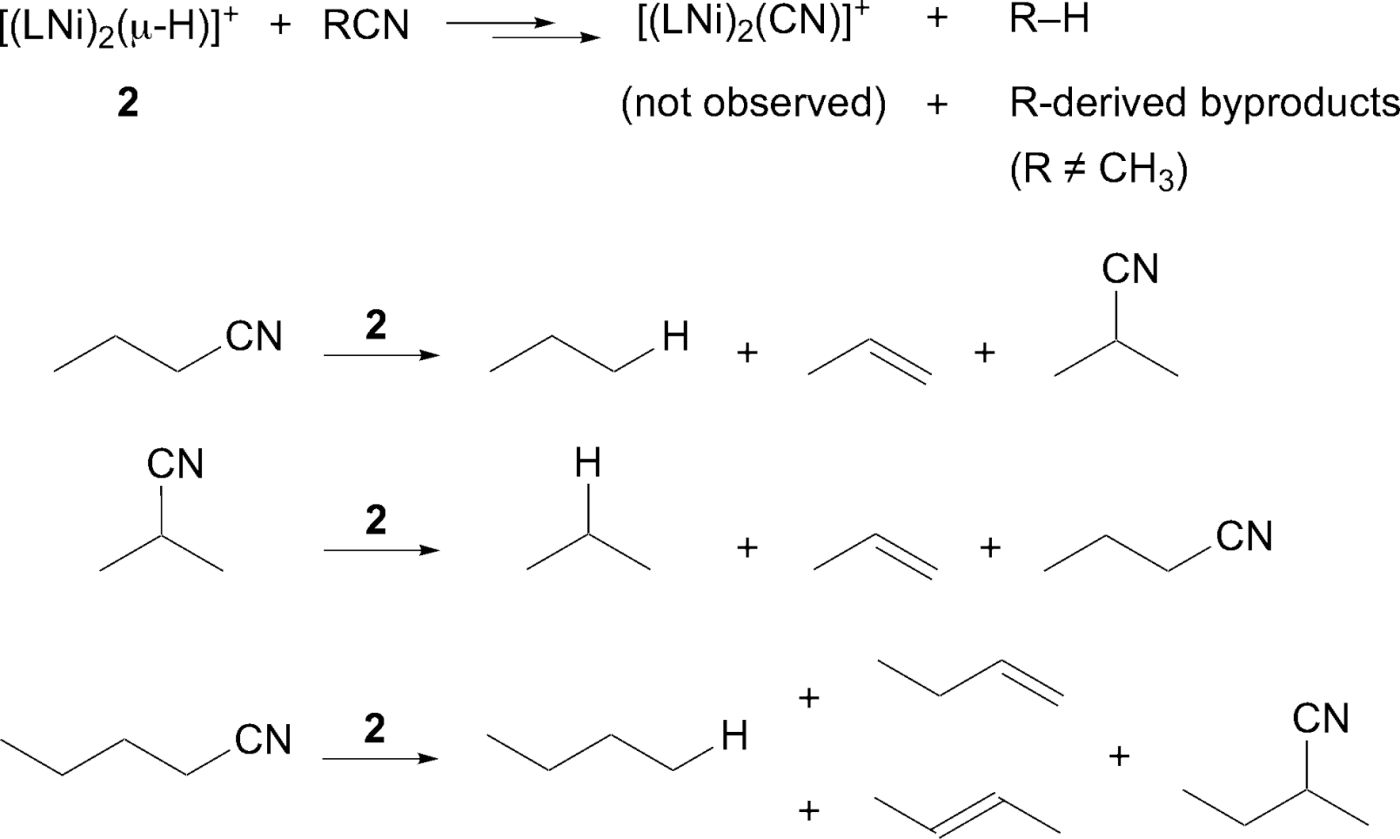
Organic Products of C–CN Bond Cleavage by 2

**Figure 5:**
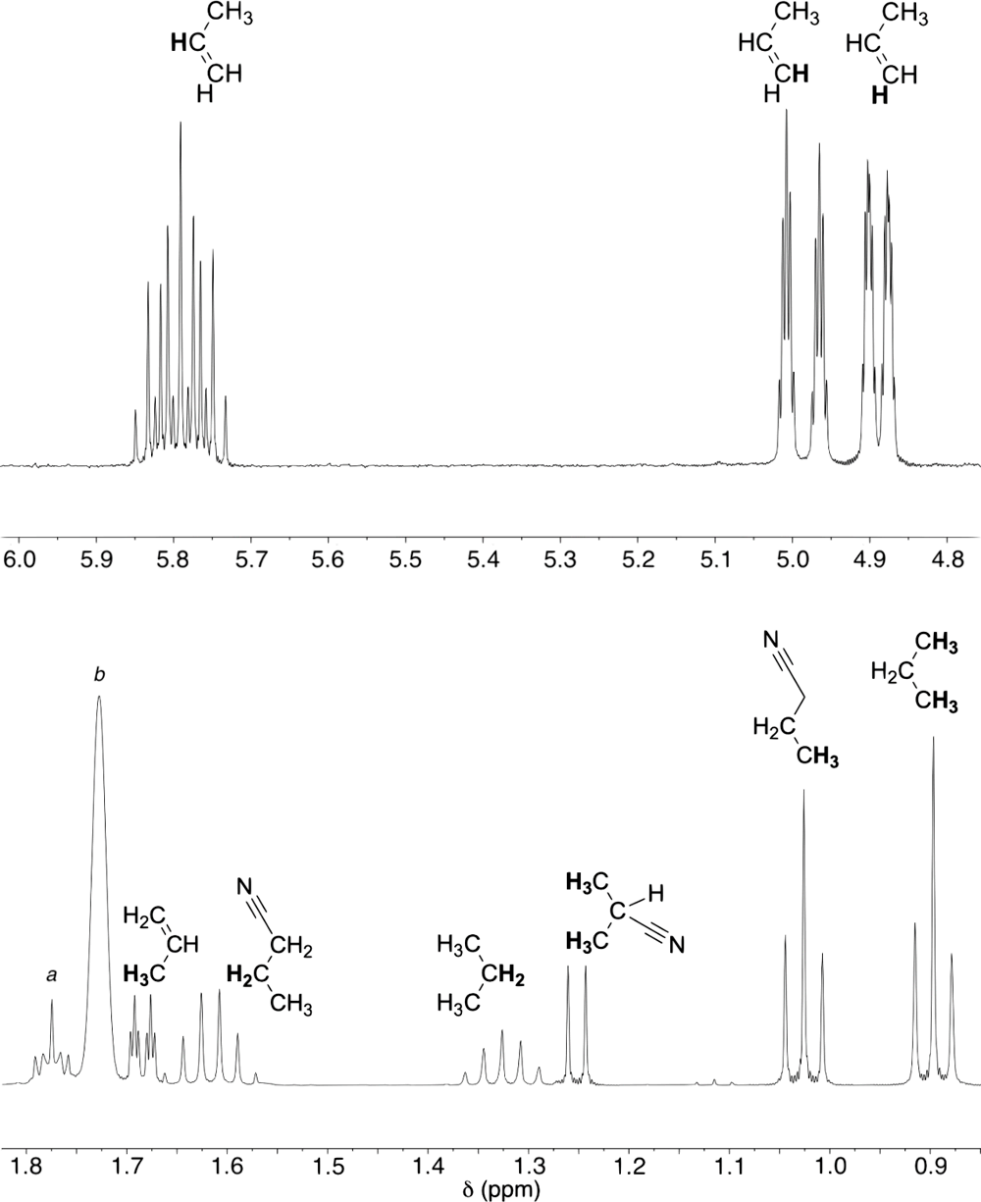
^1^H NMR signals for volatile products of the reaction between [**2**]OTf and *n*-butyronitrile in THF-*d*_8_ solution. Bold H represents the source of each resonance; adjacent H represents those involved in dipolar coupling. *a*: trace THF; *b*: THF-*d*_7_. Full spectrum is given in the Supporting Information, Figure S13.

The reaction of [**2**]OTf with longer-chain nitriles gave rise to mixtures of organic products (Scheme 3). As identified by ^1^H NMR spectroscopy, these products included alkane and alkenes,^47^ plus unchanged and isomerized nitriles.^48^ Because the nickel-derived byproducts gave rise to complex spectra, each reaction mixture was transferred under vacuum into a sealable NMR tube, then its spectrum was acquired. With *n*-butyronitrile as substrate the volatile components include propane, propene, *n*-butyronitrile, and isobutyronitrile, as shown in Figure 5. The reaction of [**2**]OTf with isobutyronitrile likewise gives rise to a mixture of propane, propene, unchanged isobutyronitrile, and *n*-butyronitrile. Valeronitrile gives *n*-butane, unchanged and isomerized nitrile, a trace of 1-butene, plus *trans*-2-butene. The predominance of internal alkenes implies that 1-butene, formed analogously to propene, readily undergoes reinsertion and β-hydride elimination to form the more stable internal alkene.^2a^^,^^49^

**Scheme 4:**
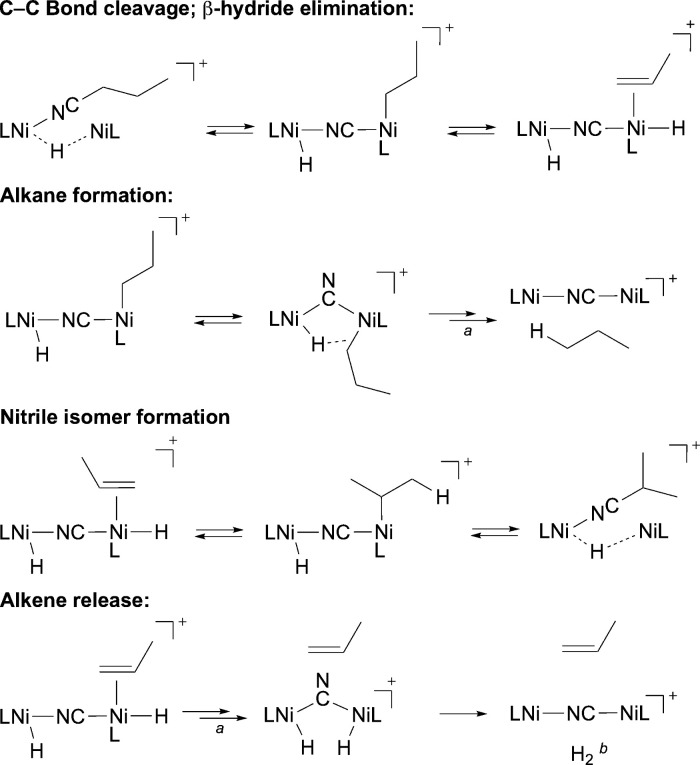
Proposed Pathways for C–CN Bond Cleavage by 2 and Subsequent Nickel Alkyl Reactions Isomerization about Ni, and between μ^2^- and μ^1^-CN, is assumed here to be rapid. H_2_ not detected but inferred from alkene formation.

The presence of alkene and of isomerized nitrile could occur through a sequence of established organometallic reactions following C–CN bond cleavage; Scheme 4 depicts key steps in this proposed sequence. Reductive elimination of a C–H bond from the dinickel(*n*-propyl) hydride intermediate would release propane. Alternatively, β-hydride elimination from the nickel-bound *n*-propyl group would form a nickel(propene) hydride moiety. Rotation of propene with respect to the nickel, followed by 1,2-insertion into the hydride, would form an isopropylnickel species. Reductive elimination of the C–CN bond would form the isomeric nitrile. In a competitive pathway, the nickel(propene) hydride intermediate could release free propene. Although H_2_ was not observed directly, its formation is implied by the formation of both propene and nickel cyanides from an aliphatic nitrile and a nickel hydride.

## Conclusions

A hydride-bridged dinickel cation, formally nickel(I), has been isolated and characterized as its triflate salt. Like many hydride-bridged dinuclear complexes, this one features a bent M–H–M moiety, but the Ni···Ni distance is too long to suggest a direct metal–metal bond. We believe the diamagnetism of this complex, reflected by its sharply defined ^1^H NMR spectral features, is best explained in terms of a three-center, four-electron bonding arrangement.

In contrast to a dicopper analogue, the cationic dinickel(I) hydride exhibits no obvious nucleophilic character. Although synthesized most cleanly from a dinickel(I) precursor and a silane, the [Ni_2_(μ-H)]^+^ core may be generated from a nickel(0) dimer plus a proton source, and subsequent treatment with strong base regenerates the nickel(0) dimer.

Robust in itself, the [Ni_2_H]^+^ core fragments and disproportionates on reaction with substrates of interest. Carbon monoxide induces the formation of (NHC)Ni(CO)_3_, Ni(CO)_4_, and imidazolium ion. Aliphatic nitriles undergo decyanation. The presumed [Ni_2_(μ-CN)]^+^ product, not observed, disproportionates to form nickel(II) cyanide oligomers and a [Ni^+^/Ni^0^] cation described previously. Acetonitrile is converted to methane in this reaction; nitriles such as butyronitrile form a mixture of alkane, alkene, and isomerized nitrile. The formation of these products suggests the involvement of nickel alkyl intermediates. Although short-lived, these intermediates persist long enough to permit chain-walking and C–CN bond reductive elimination.

## Experimental Section

### Materials

[(IDipp)Ni(μ-OCH_2_*t*-Bu)(μ^2^-O_3_SCF_3_)Ni(IDipp)]^28^ and [(IDipp)Ni(μ-Cl)(Cl)]_2_^50^ were prepared according to literature procedures, and characterized by ^1^H NMR spectroscopy. Pentamethyldisiloxane (Gelest, 97%), trimethylsilyl cyanide (Sigma-Aldrich, 98%), Isobutyronitrile (Oakwood, 99%), *n*-butyronitrile (Oakwood, 99+%), and valeronitrile (Oakwood, 98%) were degassed and handled under an atmosphere of dry nitrogen. Sodium metal (Alfa Aesar), benzophenone (Alfa Aesar), nitrogen (Airgas, industrial grade), and argon (Airgas, industrial grade) were used as received.

Tetrahydrofuran-*d*_8_ (Cambridge Isotope Laboratories) was dried over sodium benzophenone ketyl, degassed by several freeze–pump–thaw cycles, and vacuum-transferred into a resealable flask. Acetonitrile-*d*_3_ (Cambridge Isotope Laboratories) and dichloromethane-*d*_2_ were dried over calcium hydride, degassed by several freeze–pump-thaw cycles, and vacuum-transferred into a resealable flask.

Further general considerations are described in the Supporting Information.

### Spectroscopic Measurements

^1^H, ^13^C, and ^19^F spectra were obtained using Bruker Avance IIIHD 700 MHz, Bruker Avance IIIHD 500 MHz, and Bruker Avance III 400 MHz spectrometers. ^1^H and ^13^C NMR chemical shifts are referenced with respect to solvent signals and reported relative to tetramethylsilane. ^19^F NMR chemical shifts were externally referenced to neat trifluoromethylbenzene (−63.72 ppm). Infrared spectra were collected using microcrystalline samples on a Shimadzu IRAffinity-1S FT-IR Spectrophotometer equipped with an attenuated total reflection (ATR) attachment. Samples were exposed to air as briefly as possible prior to data collection.

**Elemental analyses** were performed by Atlantic Microlab, Inc. in Norcross, GA and by Robertson Microlit Laboratories, in Ledgewood, NJ.

#### Synthesis of {[(IDipp)Ni]_2_(μ-H)}[OTf] ([**2**]OTf)

A scintillation vial was charged with [(IDipp)Ni(μ-OCH_2_*t*-Bu)(μ_2_-O_3_SCF_3_)Ni(IDipp)] (**1**, 0.247 g; 0.219 mmol), THF (4 mL), and a stirbar. Pentamethyldisiloxane (0.043 mL, 0.032 g, 0.22 mmol) was added dropwise to the solution, with stirring. The vial was capped, and the reaction mixture allowed to stir for 2 h. During this time the solution changed from yellow to dark red in color. The stirbar was removed and the THF solution was layered with toluene (16 mL). The vial was capped and placed in the glovebox freezer (−35 °C) for 24 h. The resulting solid was collected on a frit and triturated with hexanes. The product was collected from the frit and dried in vacuo for 2 h, affording {[(IDipp)Ni]_2_(μ-H)}[OTf] as a red solid, 0.196 g (86%). 1H NMR (400 MHz, THF-*d*_8_): δ (ppm) 7.77 (d, *J* = 1.9 Hz, 2H, NC*H*), 7.58 (t, *J* = 7.8 Hz, 2H, para–C*H*), 7.45 (d, *J* = 7.7 Hz, 4H, meta–C*H*), 7.40 (d, *J* = 1.8 Hz, 2H, NC*H*), 6.53 (d, *J* = 7.3 Hz, 4H, meta–C*H*), 4.80 (t, *J* = 7.2 Hz, 2H, para–C*H*), 2.75 (sept, *J* = 6.9 Hz, 4H, C*H*(CH_3_)_2_), 2.29 (sept, *J* = 6.8 Hz, 4H, CH(C*H*_3_)_2_), 1.49 (d, *J* = 6.9 Hz, 12H, CH(C*H*_3_)_2_),1.41 (d, *J* = 6.8 Hz, 12H, CH(C*H*_3_)_2_), 1.11 (d, *J* = 6.8 Hz, 24H, CH(C*H*_3_)_2_),–25.56 (s, 1H, Ni*H*Ni). ^13^C{^1^H} NMR (176 MHz, THF-*d*_8_): δ (ppm) 193.48 (N*C*Ni), 146.65, 137.52, 130.67, 128.73, 126.39, 124.65, 124.07, 120.12, 113.40, 108.91, 104.77, 29.31 *C*H(CH_3_)_2_), 28.79 *C*H(CH_3_)_2_), 24.54 CH(*C*H_3_)_2_), 23.21 CH(*C*H_3_)_2_), 23.11 CH(*C*H_3_)_2_); ^19^F NMR (376 MHz, THF-*d*_8_): δ (ppm)–79.80. IR: ν (cm^–1^) 2962 (s), 2927 (s), 2868 (s), 2366 (w), 2035 (w), 1452 (s), 1387 (s), 1362 (s), 1304 (s), 1267 (s), 1222 (s), 1185 (s), 1142 (s), 1059 (s), 1033 (s), 961 (w), 938 (w), 872 (s), 792 (s), 788 (s), 724 (w), 683 (w), 636 (s), 570 (s), 516 (s), 465 (s). Anal. Calcd for C_55_H_72_F_3_N_4_Ni_2_O_3_S: C 63.30, H 6.95, N 5.37; found C 63.02, H 6.81, N 5.28.

#### Synthesis of {[(IDipp)Ni]_2_(μ-H)}[NTf_2_] ([**2**]NTf_2_)

[(IDipp)Ni (benzene)] (0.090 g, 0.17 mmol) and trifluoromethanesulfonimide (0.024 g, 0.085 mmol) were dissolved in benzene (10 mL) in a 20 mL glass scintillation vial. The solution was mixed by swirling and kept at room temperature for 21 h, resulting in the formation of a reddish-brown precipitate. The mother liquor was decanted, and the solid was collected on a fritted glass filter, and then washed with pentane (3 × 1 mL) and dried in vacuo at room temperature for 16 h, affording **[2]**NTf_2_ as a reddish-brown powder, 0.082 g (82%). ^1^H NMR (400 MHz, THF-*d*_8_): δ (ppm) 7.72 (d, *J* = 1.6 Hz, 2H, NC*H*), 7.58 (t, *J* = 7.8 Hz, 2H, *para*–C*H*), 7.45 (d, *J* = 7.6 Hz, 4H, *meta*–C*H*), 7.40 (d, *J* = 1.2 Hz, 2H, NC*H*), 6.53 (d, *J* = 7.2 Hz, 4H, meta–C*H*), 4.79 (t, *J* = 7.2 Hz, 2H, para–C*H*), 2.73 (sept, *J* = 7.0 Hz, 4H, C*H*(CH_3_)_2_), 2.29 (sept, *J* = 6.8 Hz, 4H, C*H*(CH_3_)_2_), 1.49 (d, *J* = 6.8 Hz, 12H, CH(C*H*_3_)_2_), 1.41 (d, *J* = 6.8 Hz, 12H, CH(C*H*_3_)_2_), 1.11 (d, *J* = 6.8 Hz, 24H, CH(C*H*_3_)_2_),–25.31 (s, 1H, Ni*H*Ni); ^13^C{1H} NMR (176 MHz, THF-*d*_8_): δ (ppm) 193.56 (N*C*Ni), 146.63, 137.45, 130.71, 128.72, 126.33, 124.66, 123.83, 120.13, 113.39, 108.90, 104.76, 29.31 (*C*H(CH_3_)_2_), 28.79 (*C*H(CH_3_)_2_), 24.50 (CH(*C*H_3_)_2_), 23.18 (CH(*C*H_3_)_2_), 23.07 (CH(*C*H_3_)_2_); ^19^F NMR (376 MHz, THF-*d*_8_): δ (ppm)–76.89 (s). IR: ν (cm^–1^) 2962 (s), 2931 (s), 2869 (s), 2164 (s), 1961 (w), 1465 (s), 1387 (s), 1354 (s), 1267 (s), 1228 (s), 1187 (s), 1140 (s), 1057 (s), 963 (s), 940 (s), 872 (s), 794 (s), 763 (s), 739 (s), 687 (w), 650 (w), 617 (s), 570 (s), 512 (s), 463 (s). Note: Some runs have given rise to clean **[2]**NTf_2_; others formed **[2]**NTf_2_ containing small and varying fractions (ca. 10%) of {[(IDipp)Ni]_2_}^+^ NTf_2_^–^ as judged by ^1^H NMR spectrometry.

#### Reaction of [**2**]OTf with Carbon Monoxide

A solution of **[2]**OTf (0.012 g, 0.011 mmol) in THF-*d*_8_ (0.6 mL) was prepared in a J. Young NMR tube and degassed by three freeze–pump–thaw cycles. Carbon monoxide (1 atm) was added, the valve was sealed, and the tube inverted and set upright several times to ensure mixing. The reddish-brown color disappeared over 10 min at room temperature. ^1^H NMR (400 MHz, THF-*d*_8_): [IDippH]OTf δ (ppm) 9.77 (t, *J* = 1.8 Hz, 1H, NC*H*N), 8.26 (d, *J* = 1.8 Hz, 2H, NC*H*), 7.61 (dd, *J* = 8.4 Hz, 7.2 Hz, 2H, *para*–C*H*), 7.42 (d, *J* = 7.2 Hz, 4H, *meta*–C*H*), 2.53 (sept, *J* = 6.8 Hz, 4H, C*H*(CH_3_)_2_), 1.29 (d, *J* = 6.8 Hz, 12H, CH(C*H*_3_)_2_), 1.23 (d, *J* = 6.8 Hz, 12H, CH(C*H*_3_)_2_); [(IDipp)Ni(CO)_3_] δ (ppm) 7.46 (m, 6H, *para*–C*H* and *meta*–C*H*), 7.32 (s, 2H, NC*H*), 2.71 (sept, *J* = 6.8 Hz, 4H, C*H*(CH_3_)_2_), 1.28 (d, *J* = 6.9 Hz, 12H, CH(C*H*_3_)_2_), 1.15 (d, *J* = 6.9 Hz, 12H, CH(C*H*_3_)_2_); ^13^C{^1^H} NMR (176 MHz, THF-*d*_8_): [Ni(CO)_4_] δ (ppm) 192.40 (*C*O);^40^ [IDippH]OTf δ (ppm) 146.06 (*ortho*-*C*), 132.46 (*ipso*-*C*), 128.83 (*para*-*C*), 127.54 (N*C*H), 125.19 (*meta*-*C*), 29.74 (*C*H(CH_3_)_2_), 24.47 (CH(*C*H_3_)_2_), 23.68 (CH(*C*H_3_)_2_); [(IDipp)Ni(CO) _3_] d (ppm) 198.00 (N*C*Ni), 196.24 (*C*O), 146.51 (*ortho*-*C*), 138.44 (*ipso-C*), 130.24 (*para-C*), 128.72 (N*C*H), 124.35 (*meta-C*), 29.11 (*C*H(CH_3_) _2_), 26.20 (CH(*C*H_3_)_2_), 22.74 (CH(*C*H_3_)_2_); ^19^F NMR (376 MHz, THF-*d*_8_): δ (ppm)–77.47 (s). The solution was concentrated in vacuo, taken up in C_6_D_6_, and filtered to remove [IDippH]OTf. The ^1^H NMR resonances of the resulting solution matched those reported for (IDipp)Ni(CO)_3_.^51^

***Note:****The byproduct Ni(CO)*_*4*_*is volatile and highly toxic. This reaction resulted in the generation of milligram quantities in solution, in a sealed tube. The contents were carefully destroyed afterward in a well-ventilated fume hood. All users must be aware of its hazards, plan reactions to avoid inhalation or direct contact, and plan for complete and safe disposal of residues.*

#### Deprotonation of {[(IDipp)Ni]_2_(μ-H)}[OTf] by Hexamethyldisilazide

A scintillation vial was charged with {[(IDipp)Ni]_2_(μ-H)}[OTf] (0.025 g; 0.024 mmol), a stirbar, and THF (1 mL). NaN(SiMe_3_)_2_ (0.11 M in Et_2_O, 0.22 mL, 0.024 mmol) was added dropwise to the stirred solution. The reaction vial was capped and left stirring for 1 h. The solvent was removed in vacuo. The solid was dissolved in C_6_D_6_ and filtered through a Celite pipet filter. The solvent was removed in vacuo, affording [(IDipp)Ni]_2_^23^ as a red solid, 0.017 g (81%). ^1^H NMR (400 MHz, C_6_D_6_): δ (ppm) 7.36 (t, *J* = 7.7 Hz, 2H, *para*–C*H*), 7.27 (d, *J* = 7.6 Hz, 4H, *meta*–C*H*), 6.66 (d, *J* = 1.8 Hz, 2H, NC*H*), 6.39 (d, *J* = 1.8 Hz, 2H, NC*H*), 5.72 (d, *J* = 6.4 Hz, 4H, *meta*–C*H*), 5.22 (t, *J* = 6.3 Hz, 2H, *para*–C*H*), 3.11 (sept, *J* = 6.8 Hz, 4H, C*H*(CH_3_)_2_), 2.72 (sept, *J* = 6.8 Hz, 4H, C*H*(CH_3_)_2_), 1.45 (apparent t, *J* = 6.8 Hz, 24H, CH(C*H*_3_)_2_), 1.11 (apparent t, *J* = 6.7 Hz, 24H, CH(C*H*_3_)_2_).

#### General Procedure for the Reaction of {[(IDipp)Ni]_2_(μ-H)}[OTf] with Nitriles

A 2 mL round-bottom Schlenk flask was charged with **[2]**OTf (0.040 g, 0.038 mmol), a stirbar, and THF-*d*_8_ (1 mL). Nitrile (3–5 equiv) was added, and the flask was quickly sealed. The reaction mixture was heated at 60 °C in an oil bath and allowed to stir (6 h for CH_3_CN and CD_3_CN, 24 h for longer-chain nitriles). Volatile products and solvent were then transferred in vacuo from the reaction vessel into a J. Young NMR tube.

#### [(IDipp)Ni(CN)(μ-CN)]_4_ (**3**)

A scintillation vial was charged with [(IDipp)Ni(μ-Cl)(Cl)]_2_ (0.061 g; 0.059 mmol), a stirbar, and benzene (3 mL). Trimethylsilyl cyanide (0.12 mL; 0.96 mmol) was added dropwise to the stirred reaction mixture. The vial was capped, and the mixture allowed to stir for 1 h. Over this time, the solution turned from purple to colorless. The solution was filtered through a Celite pipet filter, then concentrated in vacuo. ^1^H NMR (500 MHz, CD_2_Cl_2_): δ (ppm) 7.58 (t, *J* = 7.8 Hz, 2H, *para*–C*H*), 7.42 (d, *J* = 7.8 Hz, 4H, *meta*–C*H*), 7.22 (s, 2H, NC*H*), 2.94 (sept, *J* = 6.7 Hz, 4H, C*H*(CH_3_)_2_), 1.44 (d, *J* = 6.7 Hz, 12H, CH(C*H*_3_)_2_), 1.11 (d, *J* = 6.9 Hz, 12H, CH(C*H*_3_)_2_), 0.31 (s, 9H, Si(C*H*_3_)_3_). Note: A small singlet resonance at δ 0.36 ppm is assigned to free (*H*_3_C)_3_SiCN.^48^^13^C NMR (176 MHz, CD_2_Cl_2_) δ (ppm) 171.06 (N*C*Ni), 147.04, 134.93, 130.88, 126.02, 124.74, 28.94 (*C*H(CH_3_)_2_), 26.64 (CH(*C*H_3_)_2_), 23.23 (CH(*C*H_3_)_2_), 0.57 (Si(*C*H_3_)_3_). A small resonance at δ −1.68 ppm is assigned to free (H_3_*C*)_3_SiCN.^48^ IR: ν (cm^–1^) 2966 (m), 2931 (w), 2868 (w), 2154 (s), 1465 (m), 1406 (m), 1259 (m), 848 (s), 842 (s), 804 (s), 758 (s), 713 (s), 692 (s), 482 (s). The crude solid was dissolved in THF (2 mL), layered with pentane (15 mL), and placed in the glovebox freezer (−35 °C) for 24 h. The crystallized product was collected on a frit, triturated with pentane, and dried in vacuo for 2 h to afford 3 as a colorless solid, 0.045 g (76%). The ^1^H NMR spectrum of 3, regardless of solvent and number of recrystallizations, displayed clusters of resonances in the methyl and aromatic regions of the spectrum, suggestive of the IDipp ligand in multiple, similar environments. Anal. Calcd for C_116_H_144_N_16_Ni_4_: C 69.76, H 7.27, N 11.22; found C 69.37, H 7.23, N 11.02.

***Note:****Trimethylsilyl cyanide is highly toxic, and hydrolyzes readily to release HCN. All users must be aware of its hazards, and plan reactions to avoid direct contact with the liquid, and inhalation of its vapors.*

### X-ray Diffraction Studies

Suitable crystals were mounted on a MiTeGen micromount with perfluoroether oil. Data were collected from a shock-cooled single crystal at 100(2) K on a Bruker D8 VENTURE dual wavelength Mo/Cu four-circle diffractometer with a microfocus sealed X-ray tube using a mirror optics as monochromator and a Bruker PHOTON II detector. The diffractometer was equipped with an Oxford Cryostream 800 low temperature device and used MoK_α_ radiation (λ = 0.71073 Å).

All data were integrated with SAINT and a none absorption correction using SADABS was applied.^52,53^ The structure was solved by dual methods using SHELXT and refined by full-matrix least-squares methods against *F*^2^ by SHELXL-2014 using Olex2.^54−56^ All atoms were refined with anisotropic displacement parameters. Disordered moieties were refined using bond lengths restraints and displacement parameter restraints. Crystallographic data have been deposited with the Cambridge Crystallographic Data Centre.^57^ CIF files were generated using FinalCif.^58^

#### {[(IDipp)Ni]_2_(μ-H)}[OTf] ([**2**]OTf)

Crystals suitable for X-ray diffraction were grown by diffusion of pentane vapor into a solution of {[(IDipp)Ni]_2_(μ-H)}[OTf] in THF at room temperature. A red prism-shaped crystal, 0.319 × 0.138 × 0.116 mm, was selected.

C_54_H_73_N_4_Ni_2_,CF_3_O_3_S, *M* = 1044.67, monoclinic, space group *P*12_1_/*c*_1_ (No. 14), *a* = 12.284(1), *b* = 11.0075(8), *c* = 19.2396(14) Å, β = 99.744(3)°, *V* = 2564.0(3) Å^3^, *Z* = 2, *D*_c_ = 1.353 g cm^–3^, *F*_000_ = 1110.372, MoK_α_ radiation, λ = 0.71073 Å, *T* = 100(2) K, 2θ_max_ = 68.8°, 82,299 reflections collected, 10,721 unique (*R*_int_ = 0.0735). Final GooF = 1.0801, *R*_1_ = 0.0388, *wR*_2_ = 0.0645, *R* indices based on 8086 reflections with *I* ≥ 2σ(*I*) (refinement on *F*^2^), 699 parameters, 574 restraints. Lp and absorption corrections applied, μ = 0.833 mm^–1^.

#### [(IDipp)Ni(CN)(μ-CN)]_4_ (**3**)

Crystals suitable for X-ray diffraction were grown by layering a THF solution of **3** with pentane at −35 °C, as described above. A colorless plate, 0.687 × 0.23 × 0.054 mm, was selected.

C_132_H_176_N_16_Ni_4_O_4_ (**3**·4THF). *M* = 2285.72, monoclinic, space group *P12*_*1*_/*n*_*1*_ (No. 14), *a* = 19.5394(9) Å, *b* = 15.8369(8) Å, *c* = 20.4489(9) Å, β = 104.868(4)°, *V* = 6115.9(5) Å^3^, *Z* = 2, *D*_c_ = 1.241 g cm^–3^, *F*_000_ = 2448, CuK_α_ radiation, λ = 1.54178 Å, *T* = 100(2) K, 2θ_max_ = 69.3°, 44,469 reflections collected, 10,006 unique (*R*_int_ = 0.1317). Final GooF = 1.009, *R*_1_ = 0.1050, *wR*_2_ = 0.2444, R indices based on 5142 reflections with *I* ≥ 2σ(*I*) (refinement on *F*^2^), 719 parameters, 667 restraints. Absorption correction applied, μ = 1.145 mm^–1^.

#### [(IDipp)Ni(CN)(μ-CN)]_3_ (**3′**)

Crystals suitable for X-ray diffraction were grown by diffusion of *n*-pentane vapor into a THF solution of **3** over a period of 3 days at ambient temperature. A light yellow plate, 0.319 × 0.233 × 0.133 mm, was selected.

C_99_H_132_N_12_Ni_3_O_3_(3′·3THF). *M* = 1714.29, trigonal, space group *P3* (No 147), *a* = 21.9585(5) Å, *b* = 21.9585(5) Å, *c* = 13.4498(4) Å, α = 90°, β = 90°, γ = 120°, *V* = 5616.3(3) Å^3^, *Z* = 2, *D*_c_ = 1.014 g cm^–3^, *F*_000_ = 1836, CuK_α_ radiation, λ = 1.54184 Å, *T* = 100.00(10) K, 2θ_max_ = 75.073°, 39,010 reflections collected, 7587 unique (*R*_int_ = 0.0526). Final GooF = 1.039, *R*_1_ = 0.0560, *wR*_2_ = 0.1378, R indices based on 6744 reflections with *I* ≥ 2σ(*I*) (refinement on *F*^2^), 394 parameters, and 654 restraints. Absorption correction applied, μ = 0.935 mm^–1^.

## Supporting Information Available

Further experimental details; NMR and IR spectra; and further structural information for complexes **3** and **3′** (PDF)
