# Supplementary material for: Reversible C–CN Bond Cleavage by a Formal Dinickel(I) Hydride Cation
Source: Organometallics. 2024 Nov 1;43(22):2895–905. doi: 10.1021/acs.organomet.4c00340 (PMC11600506; doi:10.1021/acs.organomet.4c00340)
Supplement: Supplementary file 1 — om4c00340_si_001.pdf [file om4c00340_si_001.pdf]

Reversible C–CN Bond Cleavage by a Formal Dinickel(I) Hydride Cation

Yu Cao[†∫], Neil A. Dodd[†‡], John Bacsa[§], Joseph P. Sadighi[†*]

[†] School of Chemistry and Biochemistry, Georgia Institute of Technology, Atlanta, Georgia 30332-0400, USA

[∫] Current address: 10x Genomics, Pleasanton, CA 94588, USA

[‡] Current address: Bakelite Synthetics, Atlanta, GA 30338, USA

[§] X-ray Crystallography Center, Department of Chemistry, Emory University, 1515 Dickey Drive, Atlanta, Georgia 30322, USA

jsadighi3@gatech.edu

Supporting Information

Contents

General Considerations S2

Experimental Spectra and Additional Procedures

{[(IDipp)Ni]$_2$(μ-H)}OTf S2–S4

{[(IDipp)Ni]$_2$(μ-H)}NTf_2 S4–S5

Deprotonation of {[(IDipp)Ni]$_2$(μ-H)}OTf by NaN($SiMe_3$)$_2$ S6

Reaction of {[(IDipp)Ni]$_2$(μ-H)}OTf with CO S6-S7

Reaction of {[(IDipp)Ni]$_2$(μ-H)}OTf with acetonitrile S7

Reaction of {[(IDipp)Ni]$_2$(μ-H)}OTf with acetonitrile-d_3 S8

Reaction of {[(IDipp)Ni]$_2$(μ-H)}OTf with n-butyronitrile S8

Reaction of {[(IDipp)Ni]$_2$(μ-H)}OTf with isobutyronitrile S9

Reaction of {[(IDipp)Ni]$_2$(μ-H)}OTf with valeronitrile S9

Generation and disproportionation of {[(IDipp)Ni]$_2$(μ-CN)}OTf. S10–S11

Reaction between [(IDipp)Ni(Cl)(μ-Cl)]$_2$ and (CH_3)$_3$SiCN S11–S13

Solid-state structure of [(IDipp)Ni(CN)(μ-CN)]$_4$ S13

Extended solid-state structure of [(IDipp)Ni(CN)(μ-CN)]$_3$ S14

References S14

General Considerations

Unless otherwise indicated, manipulations were performed in an MBraun glovebox under an inert atmosphere of nitrogen, or in sealable glassware on a Schlenk line under an atmosphere of argon. Glassware and magnetic stir bars were dried in a ventilated oven at 160°C and were allowed to cool under vacuum. Molecular sieves (Alfa Aesar) and Celite (EMD 545) were dried under vacuum for at least twelve hours at 160 °C. Tetrahydrofuran, toluene, and hexanes (EMD Millipore Omnisolv) were sparged thoroughly with ultra-high purity argon for 30 min prior to first use, dried using an MBraun solvent purification system, and stored over molecular sieves in resealable flasks in the glovebox. Benzene (Alfa Aesar, ACS grade) and pentane (Alfa Aesar, ACS grade) were dried over sodium benzophenone ketyl in a sealed flask until a purple color was achieved. They were then degassed, vacuum-transferred to a resealable flask, and stored over molecular sieves in the glovebox. Acetonitrile (Alfa Aesar, ACS grade) was transferred to a resealable flask, degassed, dried over molecular sieves (4A, beads, 8-12 mesh) for 72 h prior to first use, and stored in the glovebox.

Experimental Spectra and Additional Procedures

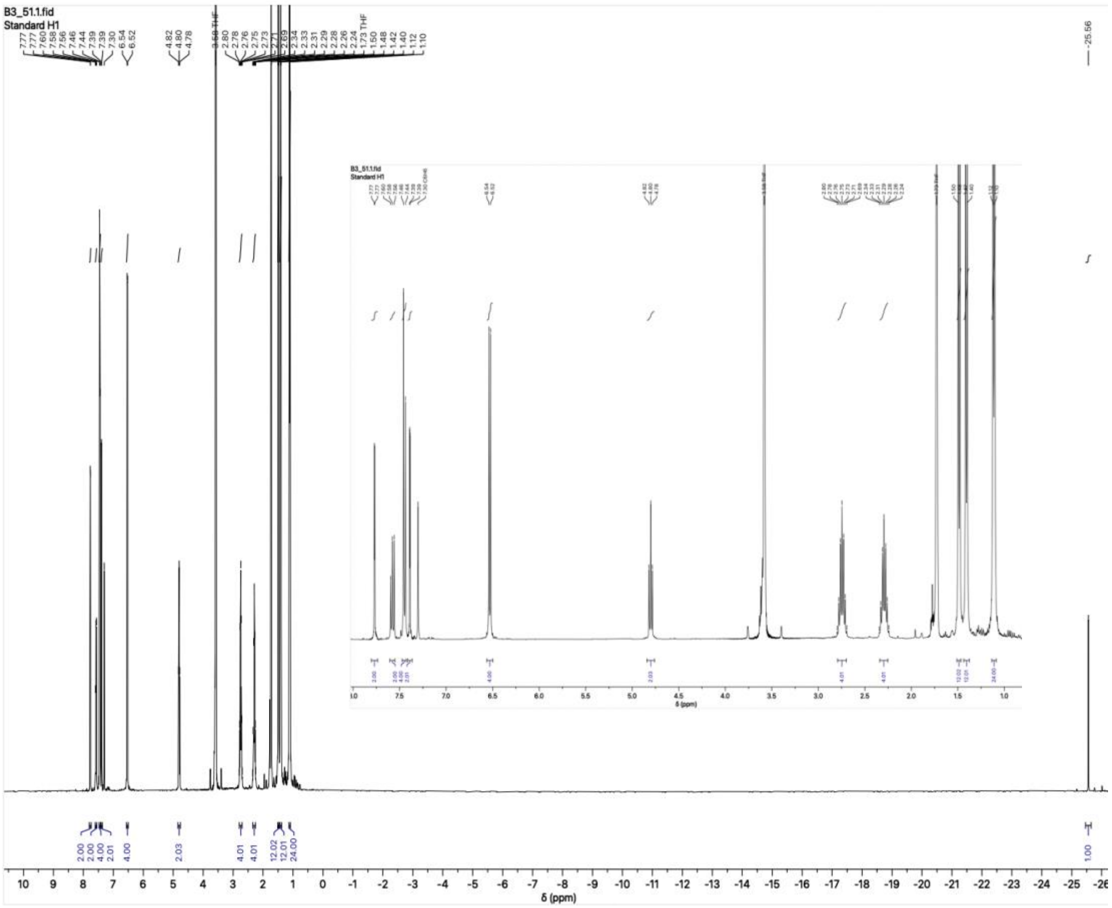

Figure S1. ^{1}H NMR spectrum of {[(IDipp)Ni]$_2$(μ-H)}OTf ([**2**]OTf) in THF-d_8 solution. A trace of benzene (δ 7.30 ppm)[1] is present as the result of benzophenone ketyl decomposition.

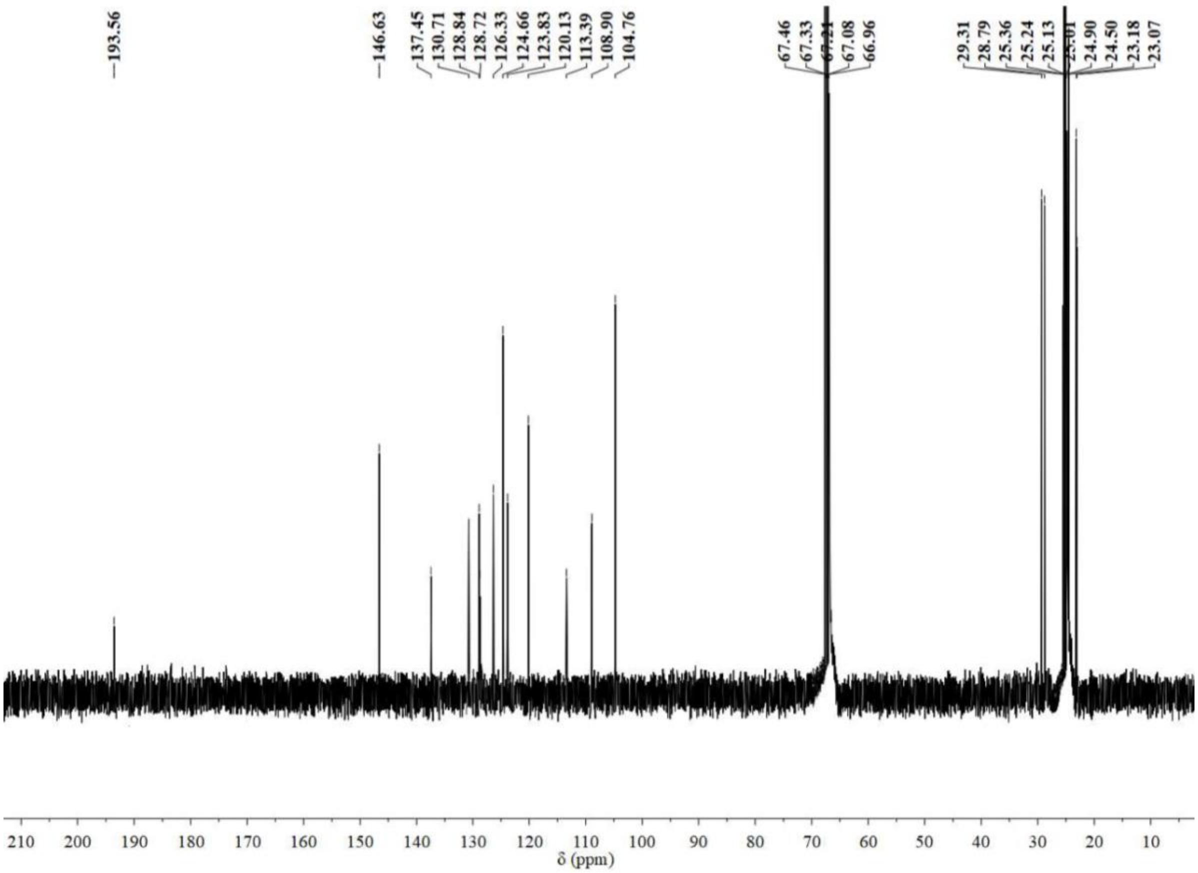

Figure S2. ${}^{13}C\{{}^{1}H\}$ NMR spectrum of $\{[(IDipp)Ni]_2(\mu\text{-}H)\}OTf$ ([**2**]OTf) in THF-d_8 solution. A trace of benzene (δ 128.84 ppm)[1] is present as the result of benzophenone ketyl decomposition.

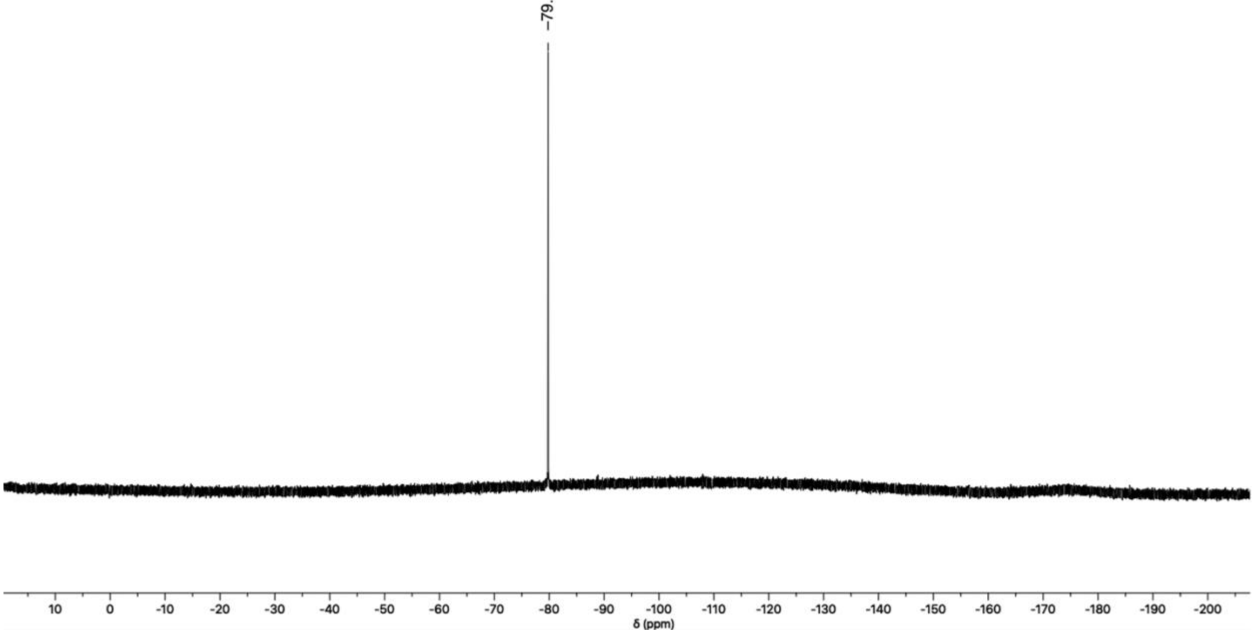

Figure S3. ${}^{19}F$ NMR spectrum of $\{[(IDipp)Ni]_2(\mu\text{-}H)\}OTf$ ([**2**]OTf) in THF-d_8 solution.

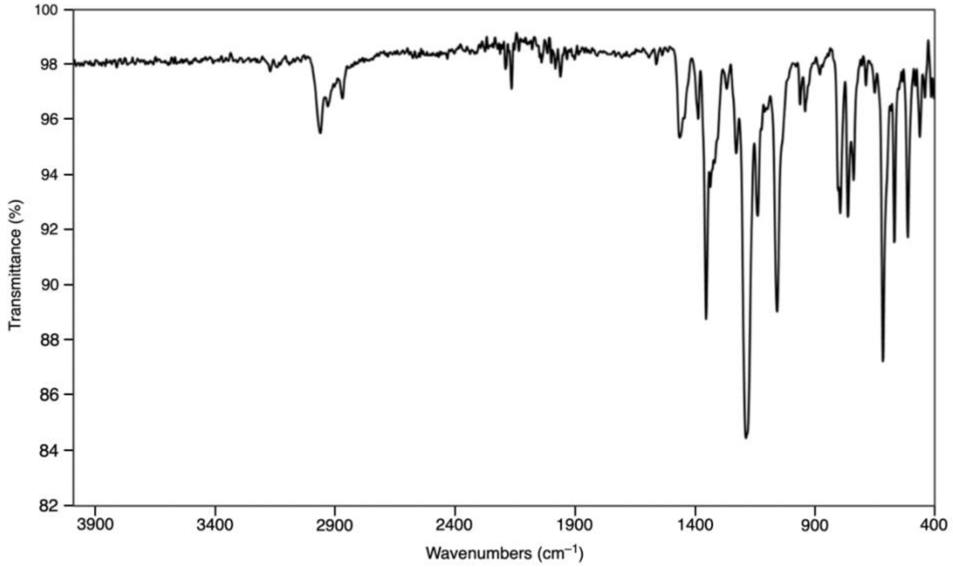

Figure S4. IR spectrum of {[(IDipp)Ni]$_2$(μ-H)}[OTf] ([**2**]OTf).

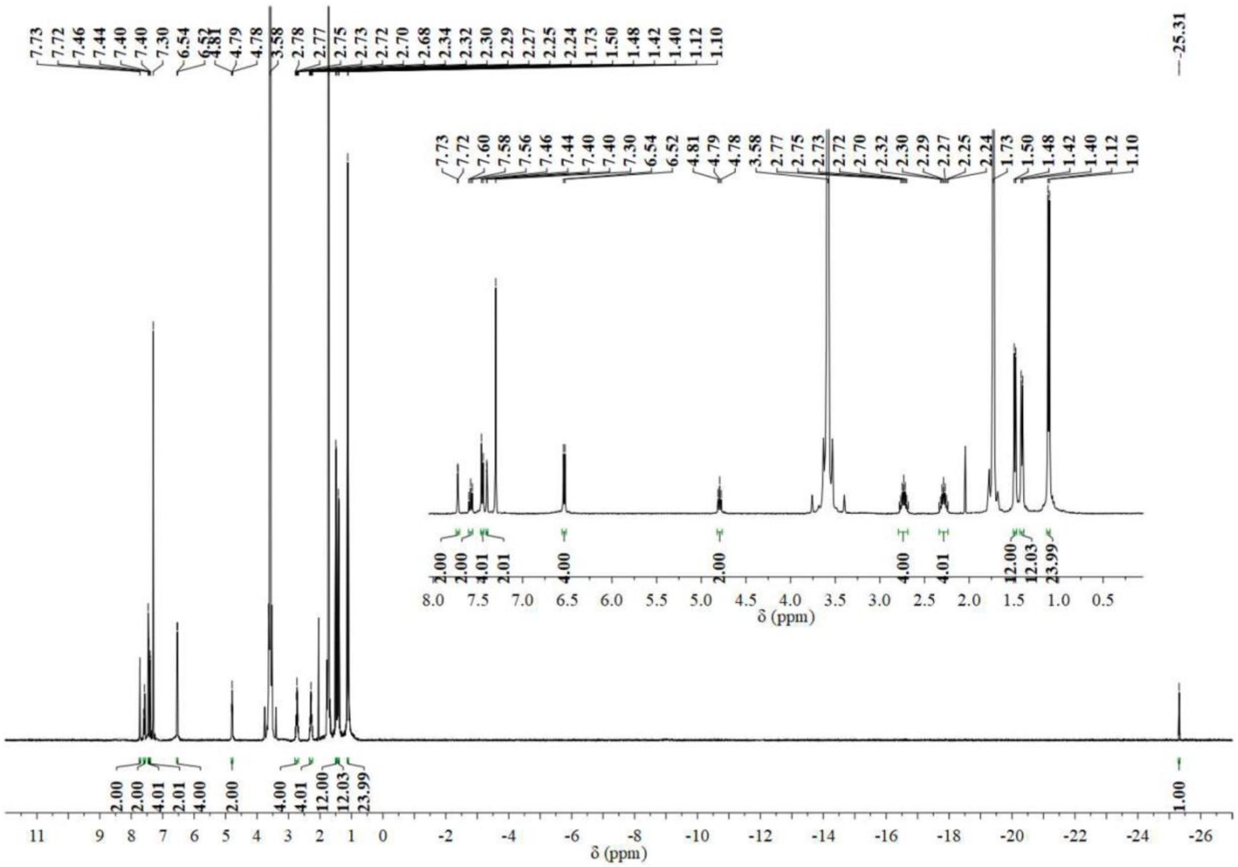

Figure S5. ^{1}H NMR spectrum of {[(IDipp)Ni]$_2$(μ-H)}NTf$_2$ ([**2**]NTf$_2$) in THF-d_8 solution. A trace of benzene (δ 7.30 ppm)[1] is present as the result of benzophenone ketyl decomposition.

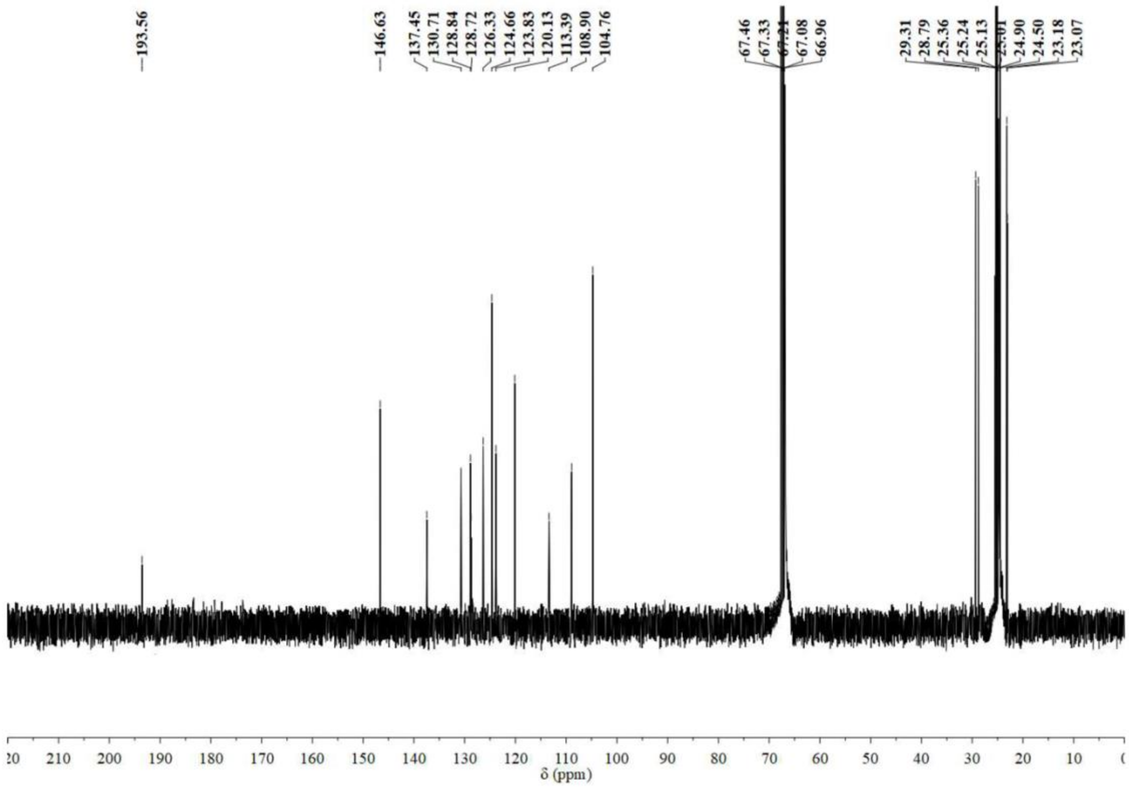

Figure S6. $^{13}C\{^{1}H\}$ NMR spectrum of $\{[(IDipp)Ni]_2(\mu\text{-}H)\}NTf_2$ ([**2**]NTf_2) in THF-d_8 solution. Trace benzene (δ 128.84 ppm)[1] is present as the result of benzophenone ketyl decomposition.

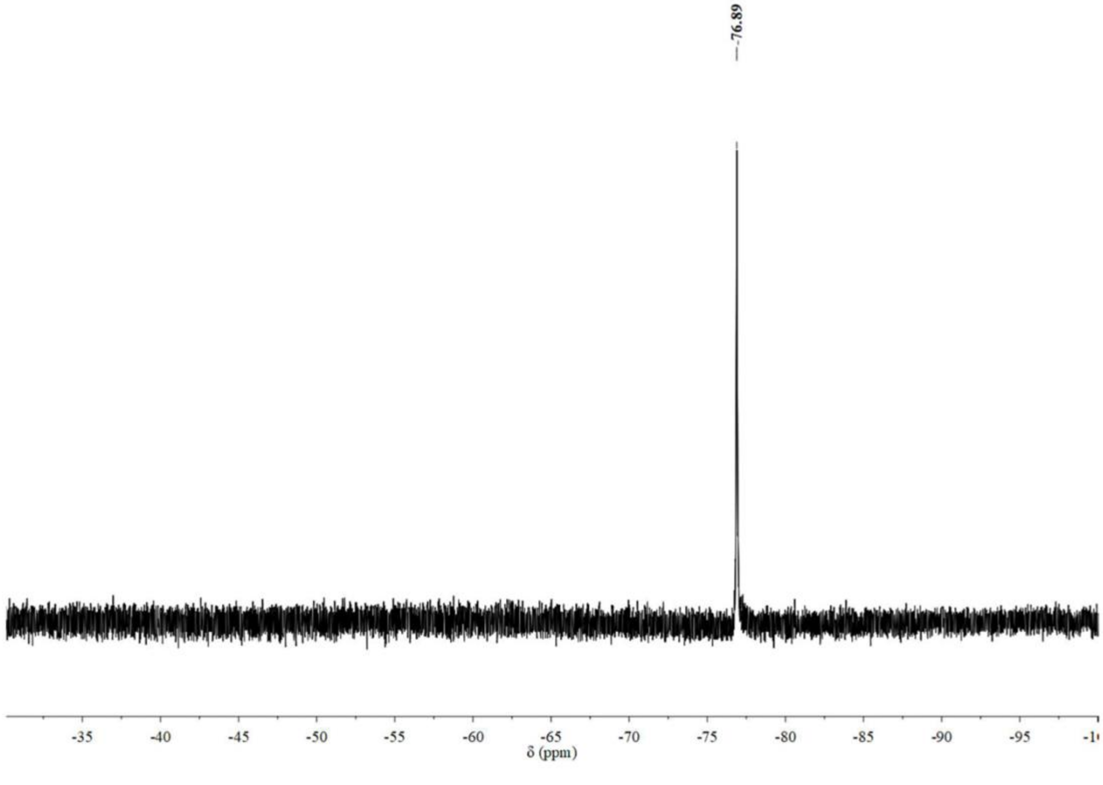

Figure S7. ^{19}F NMR spectrum of $\{[(IDipp)Ni]_2(\mu\text{-}H)\}NTf_2$ ([**2**]NTf_2) in THF-d_8 solution.

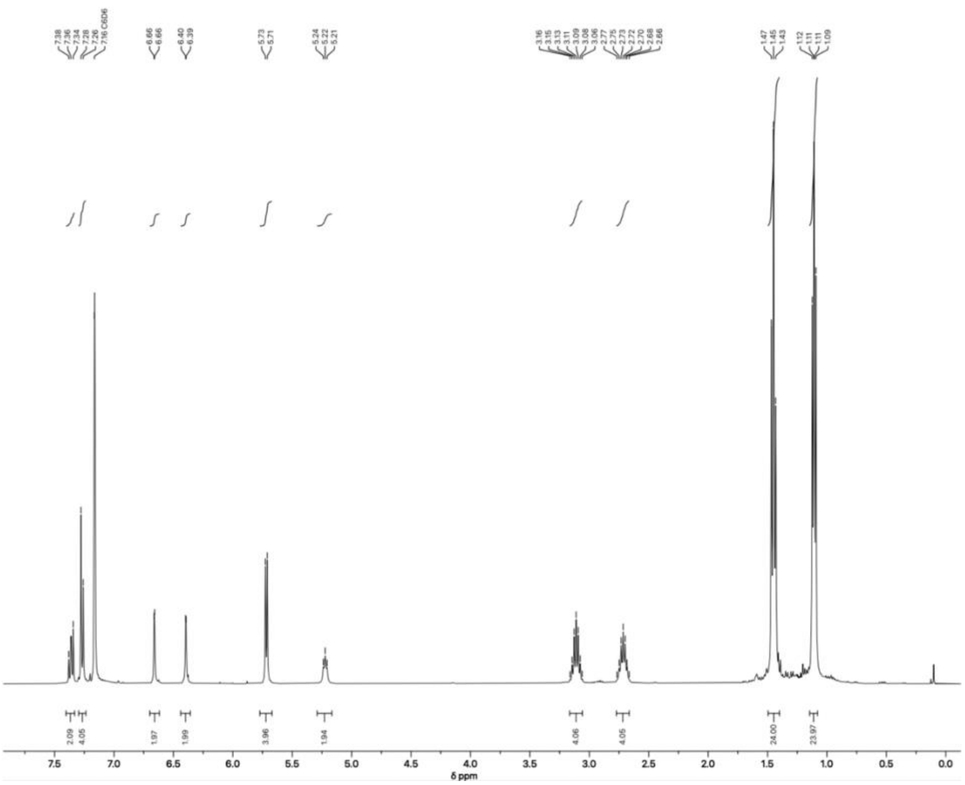

Figure S8. ^{1}H NMR spectrum of [(IDipp)Ni]$_2$,[3] formed by reaction of [**2**]OTf with NaN(SiMe$_3$)$_2$ in THF solution. Product was isolated and dissolved in C_6D_6.

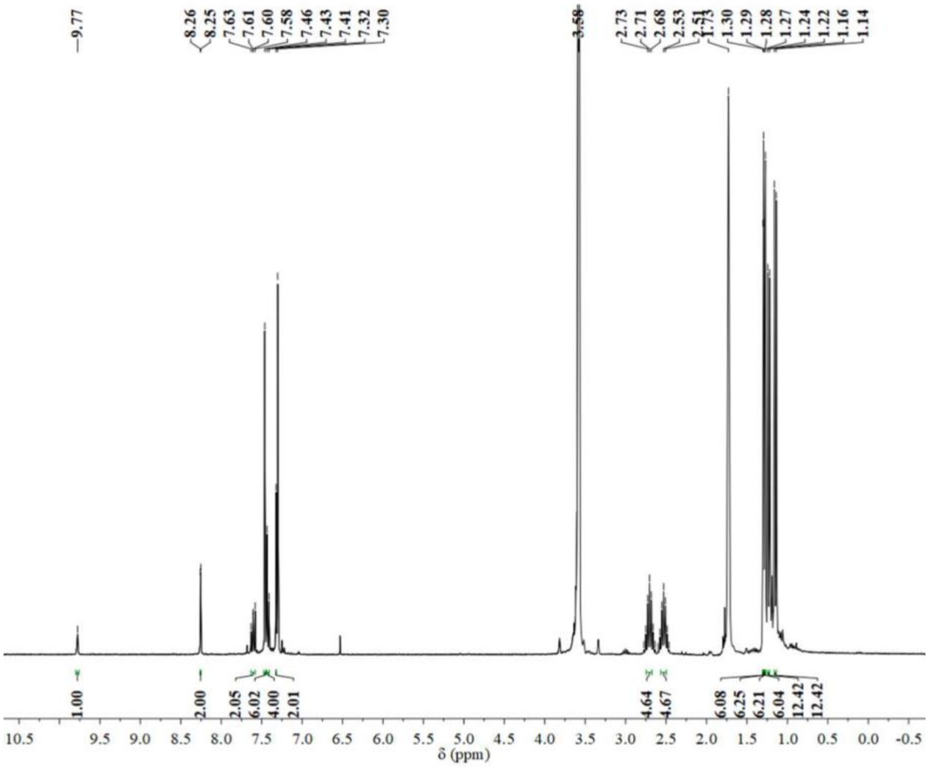

Figure S9. ^{1}H NMR spectrum for the reaction mixture formed by [**2**]OTf and CO in THF-d_8 solution. Identifiable resonances include those of [IDippH]OTf and (IDipp)Ni(CO)$_3$.

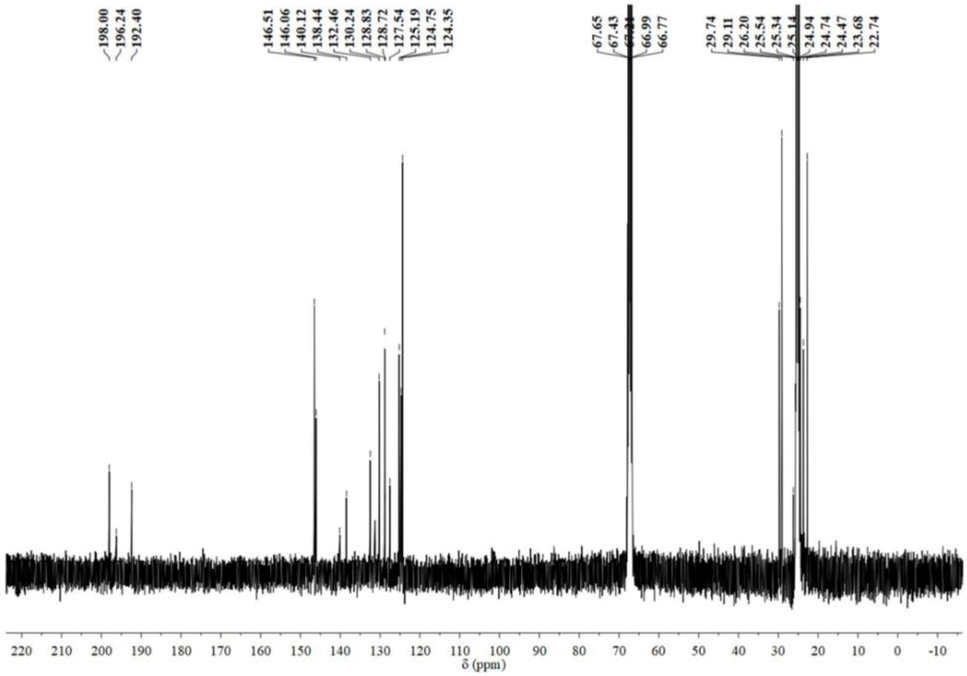

Figure S10. $^{13}C\{^{1}H\}$ NMR spectrum for the reaction mixture formed by [**2**]OTf and CO in THF-d_8 solution. A trace of benzene (δ 128.83 ppm)[1] is present as the result of benzophenone ketyl decomposition. The resonance at δ 192.40 ppm is assigned to $Ni(CO)_4$.[2]

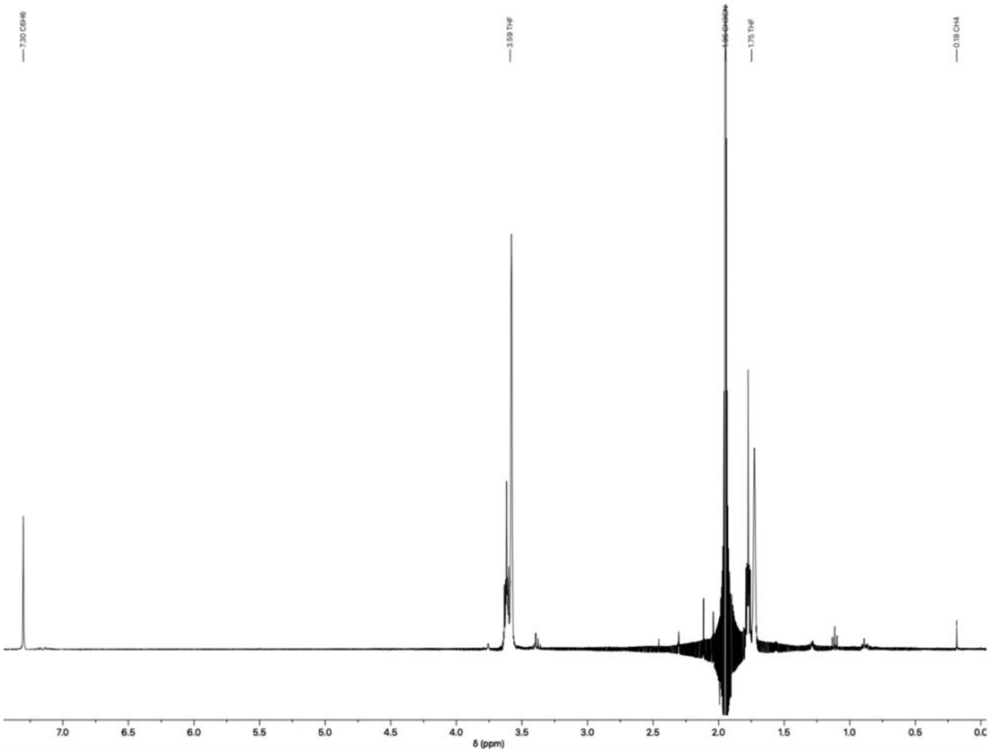

Figure S11. ^{1}H NMR spectrum of volatile products from the reaction of [**2**]OTf with CH_3CN in THF-d_8 solution, followed by vacuum transfer into a J. Young NMR tube.

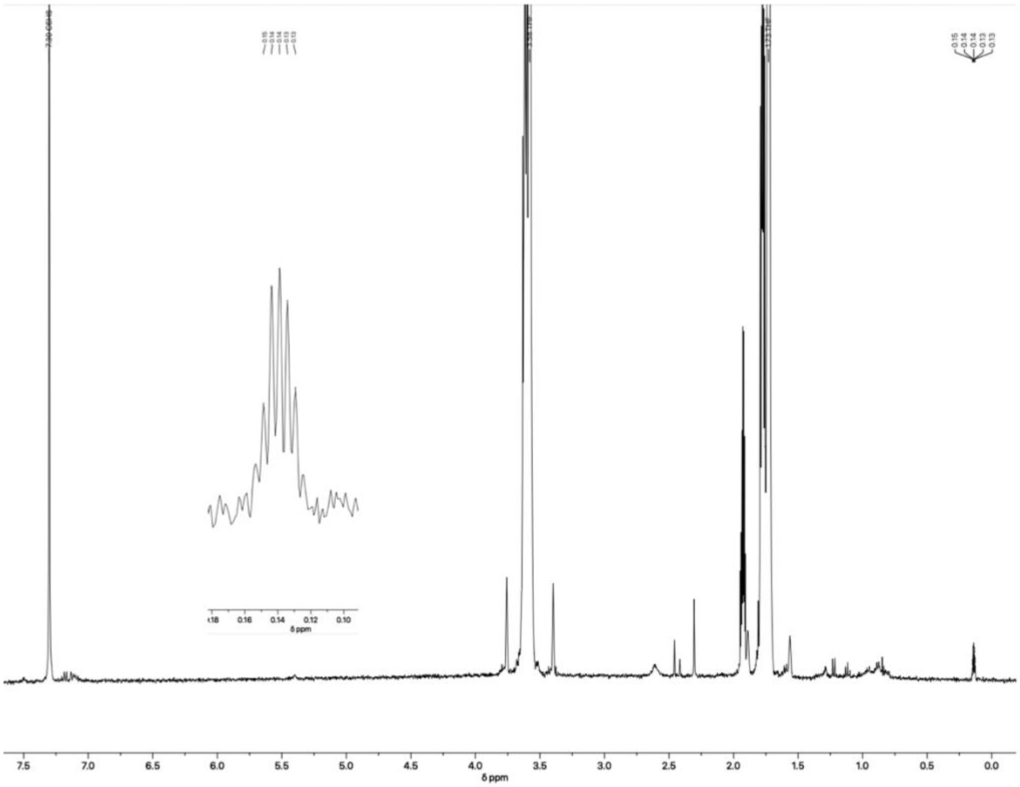

Figure S12. ^{1}H NMR spectrum of volatile products from the reaction of [**2**]OTf with CD_3CN in THF-d_8 solution, followed by vacuum transfer into a J. Young NMR tube.

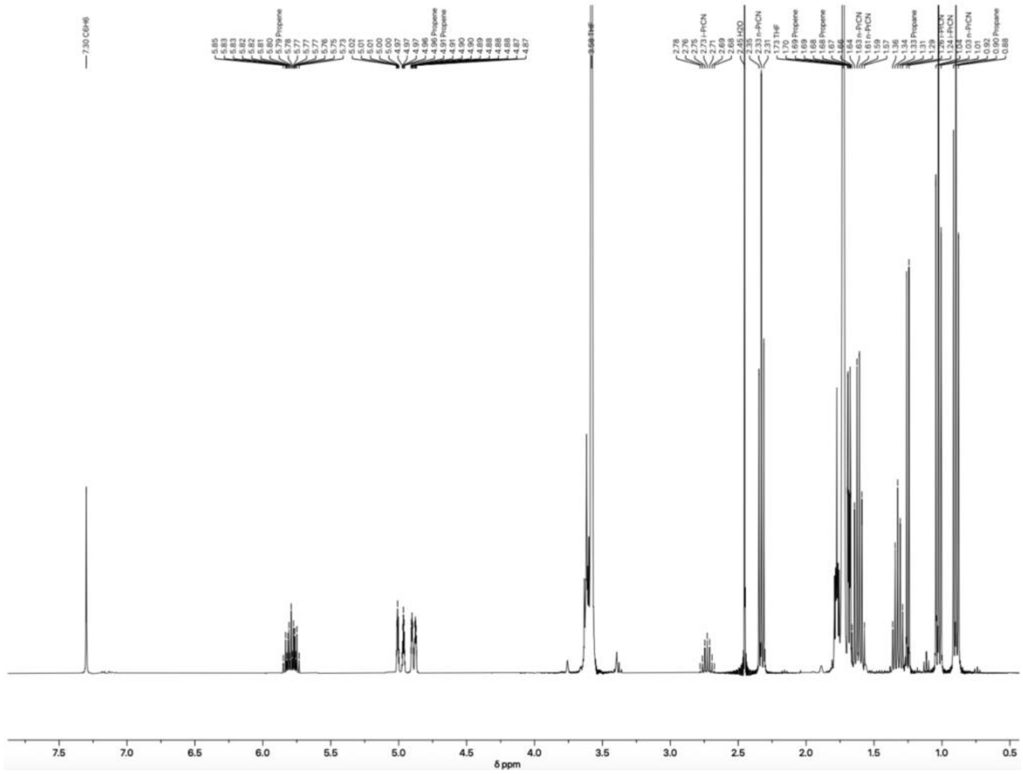

Figure S13. ^{1}H NMR spectrum of volatile products from the reaction of [**2**]OTf with *n*-butyronitrile in THF-d_8 solution, followed by vacuum transfer into a J. Young NMR tube.

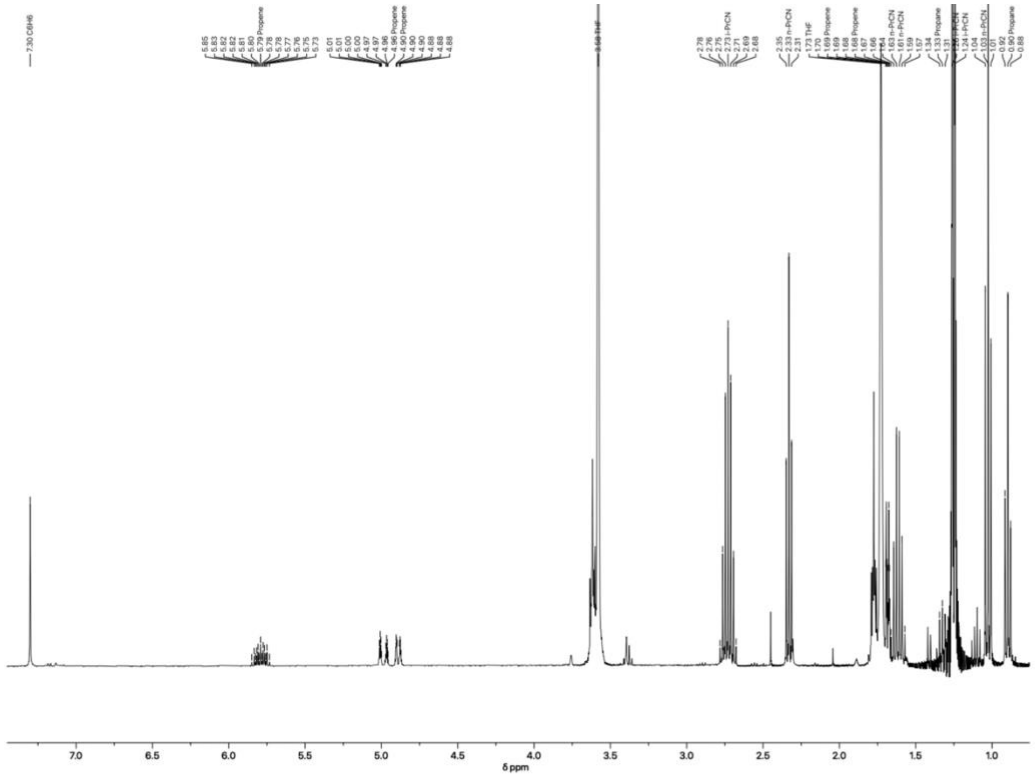

Figure S14. ^{1}H NMR spectrum of volatile products from the reaction of [**2**]OTf with isobutyronitrile in THF-d_8 solution, followed by vacuum transfer into a J. Young NMR tube.

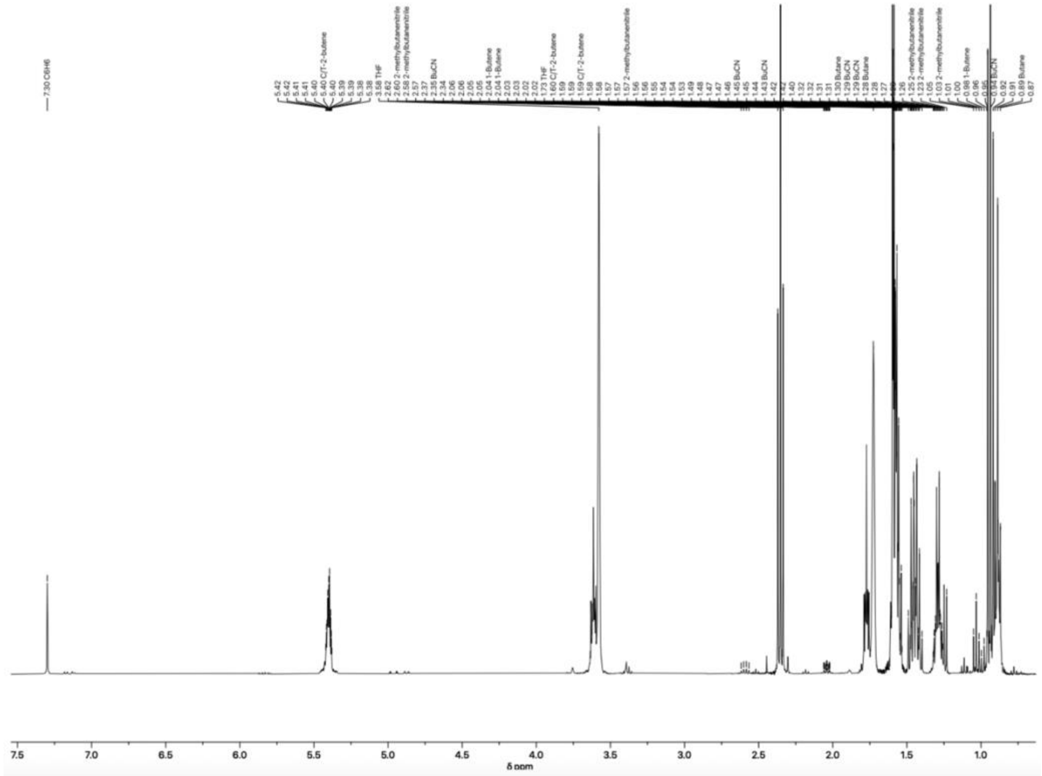

Figure S15. ^{1}H NMR spectrum of volatile products from the reaction of [**2**]OTf with valeronitrile in THF-d_8 solution, followed by vacuum transfer into a J. Young NMR tube.

Generation and disproportionation of {[(IDipp)Ni]$_2$(μ-CN)}OTf. A scintillation vial was charged with [(IDipp)Ni]$_2$(μ-ONp)(μ-OTf) (0.055 g; 0.049 mmol), a stirbar, and benzene (2 mL). Trimethylsilyl cyanide (0.16 M in benzene, 0.31 mL, 0.050 mmol) was added dropwise to the stirred solution. The vial was capped and left stirring for 1 h. Over this time, the reaction mixture turned from yellow to orange-brown in color. The mixture was filtered through a Celite pipette filter. The benzene solution was set aside, and the collected red solid was dissolved in THF. Each solution was concentrated in vacuo, and the residual solid dissolved in the corresponding deuterated solvent.

Note: *Trimethylsilyl cyanide is highly toxic, and hydrolyzes readily to release HCN. All users must be aware of its hazards, and plan reactions to avoid direct contact with the liquid, and inhalation of its vapors.*

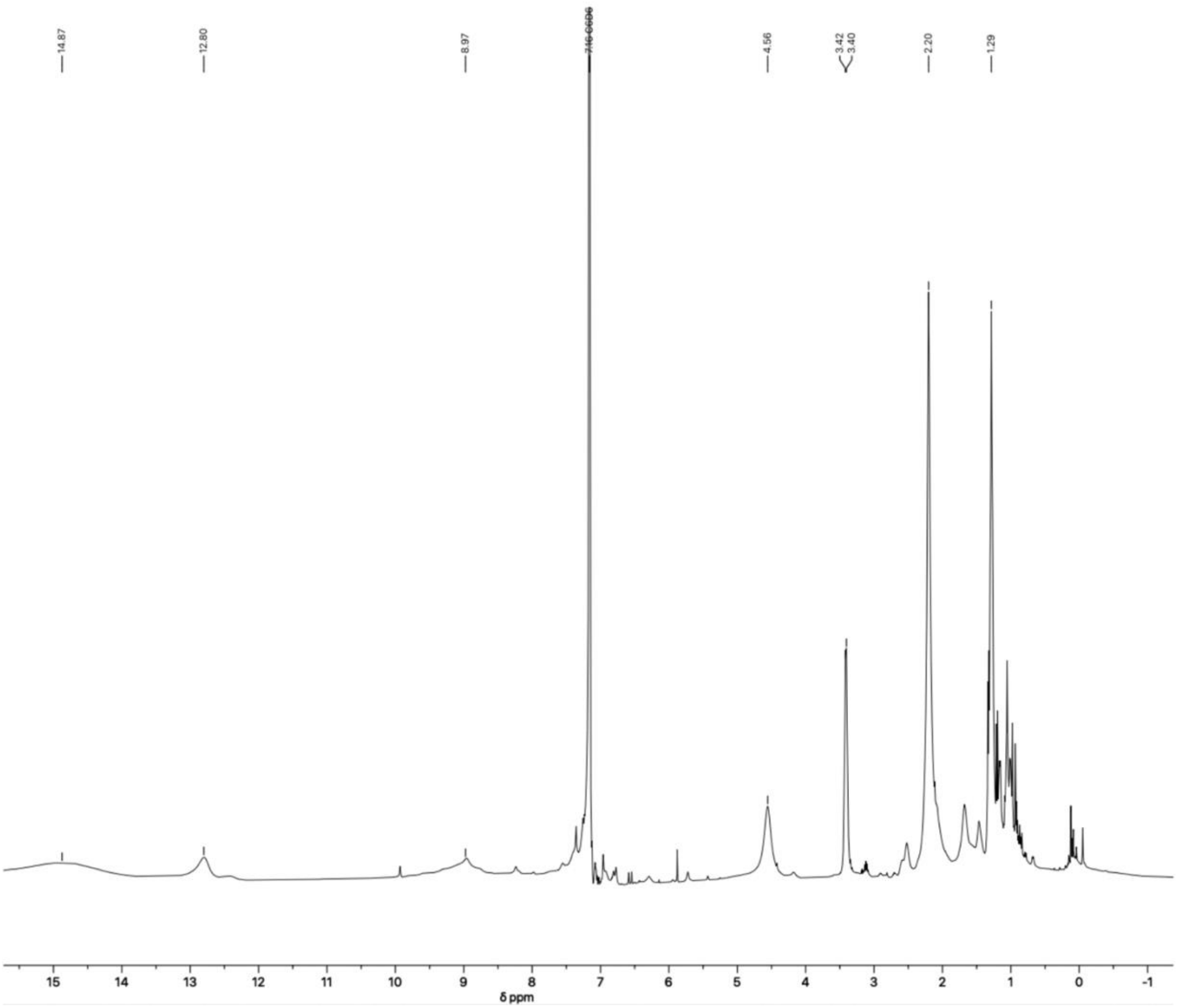

Figure S16. ^{1}H NMR spectrum of benzene-soluble products from the reaction of [(IDipp)Ni]$_2$(μ-ONp)(μ-OTf) with $(CH_3)_3SiCN$. This portion was concentrated and redissolved in C_6D_6.

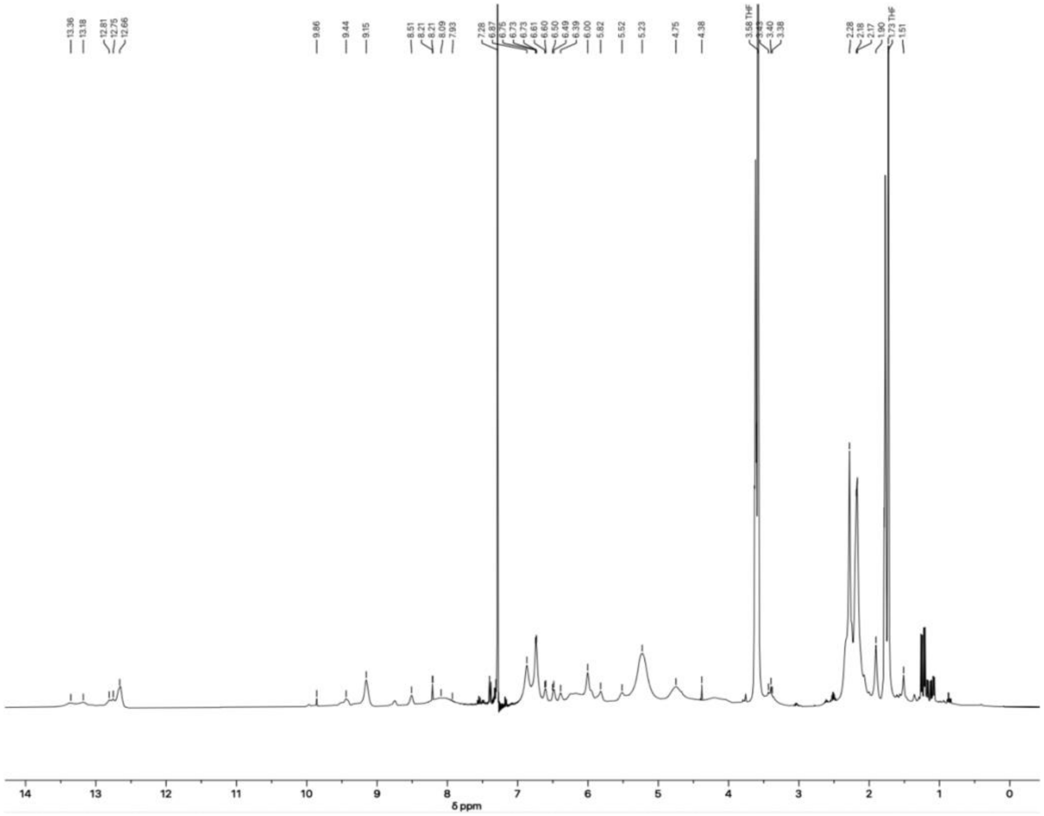

Figure S17. ^{1}H NMR spectrum of benzene-insoluble products from the reaction of $[(IDipp)Ni]_2(\mu\text{-}ONp)(\mu\text{-}OTf)$ with $(CH_3)_3SiCN$. This portion was concentrated and redissolved in THF-d_8.

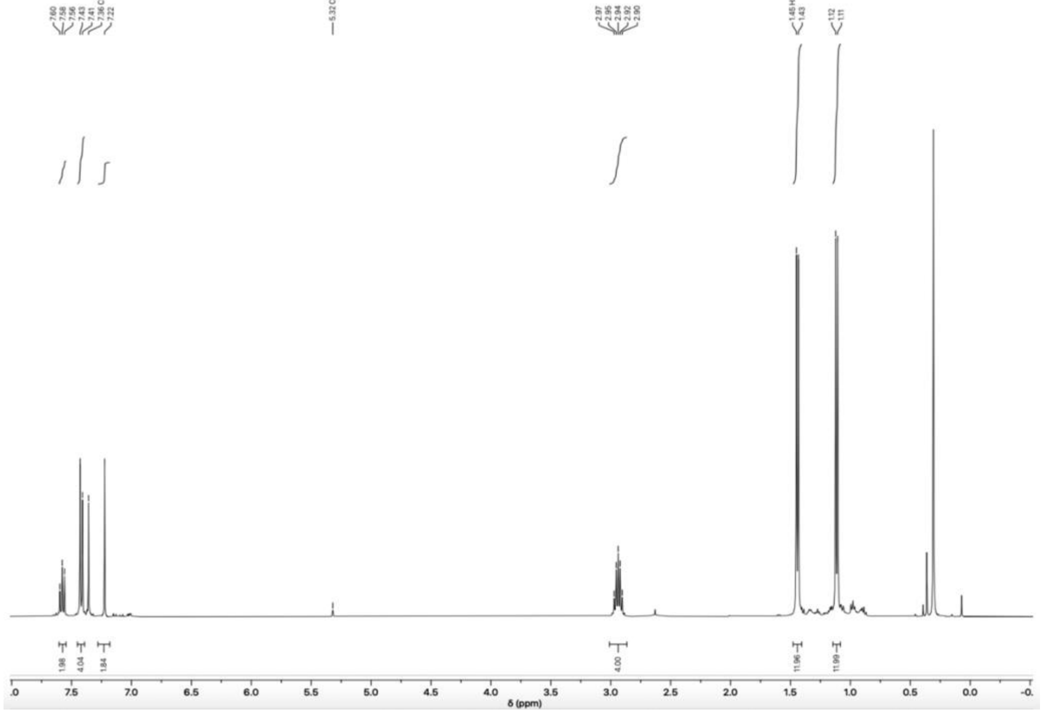

Figure S18. ^{1}H NMR spectrum of the crude product from reaction of $[(IDipp)Ni(Cl)(\mu\text{-}Cl)]_2$ with $(CH_3)_3SiCN$. Product was isolated by concentration in vacuo and dissolved in CD_2Cl_2.

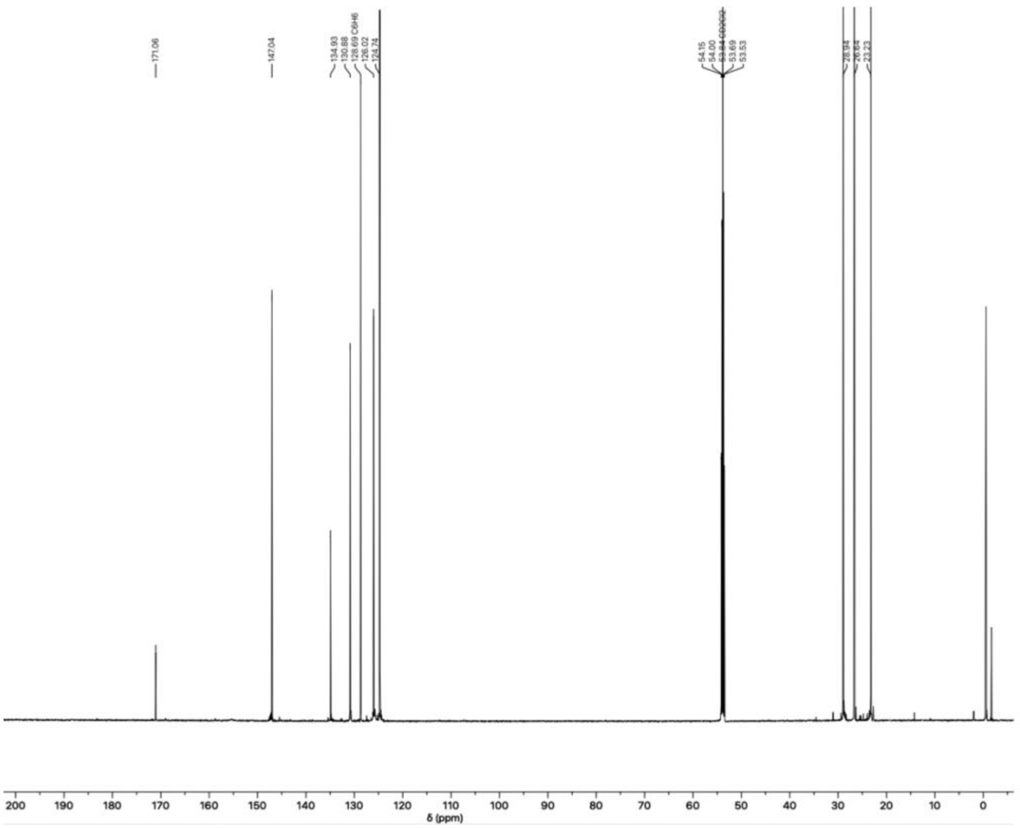

Figure S19. $^{13}C\{^1H\}$ NMR spectrum of the crude reaction product from $[(IDipp)Ni(Cl)(\mu\text{-}Cl)]_2$ and $(CH_3)_3SiCN$, in CD_2Cl_2 solution.

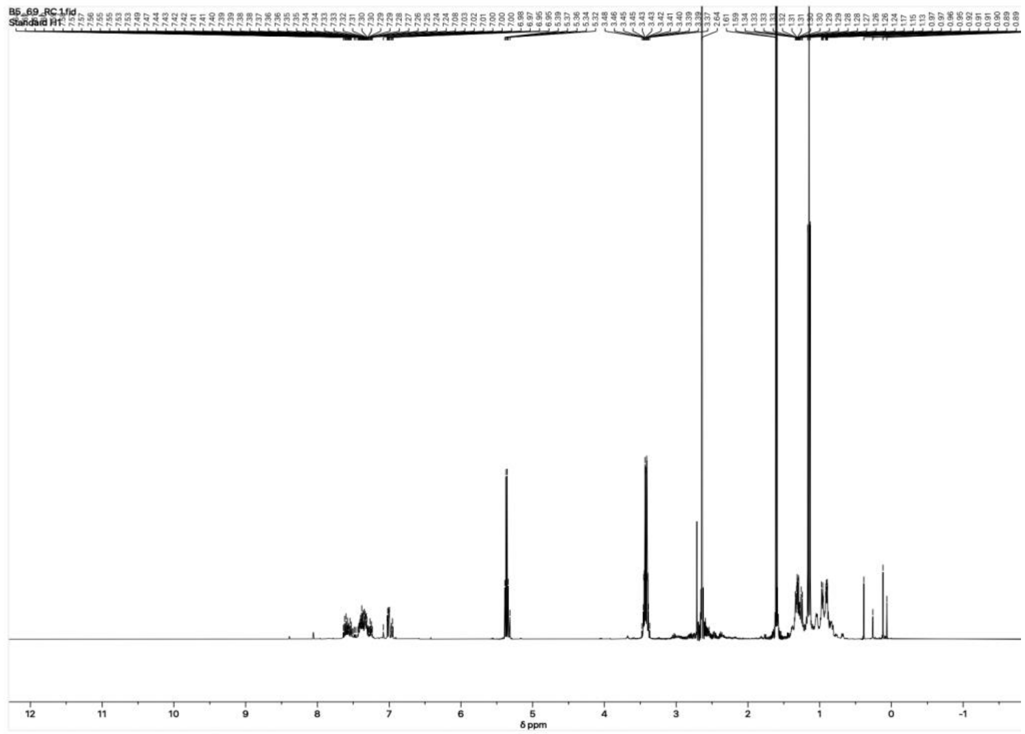

Figure S20. ^1H NMR spectrum of recrystallized $[(IDipp)Ni(CN)(\mu\text{-}CN)]_4$ in CD_2Cl_2 solution.

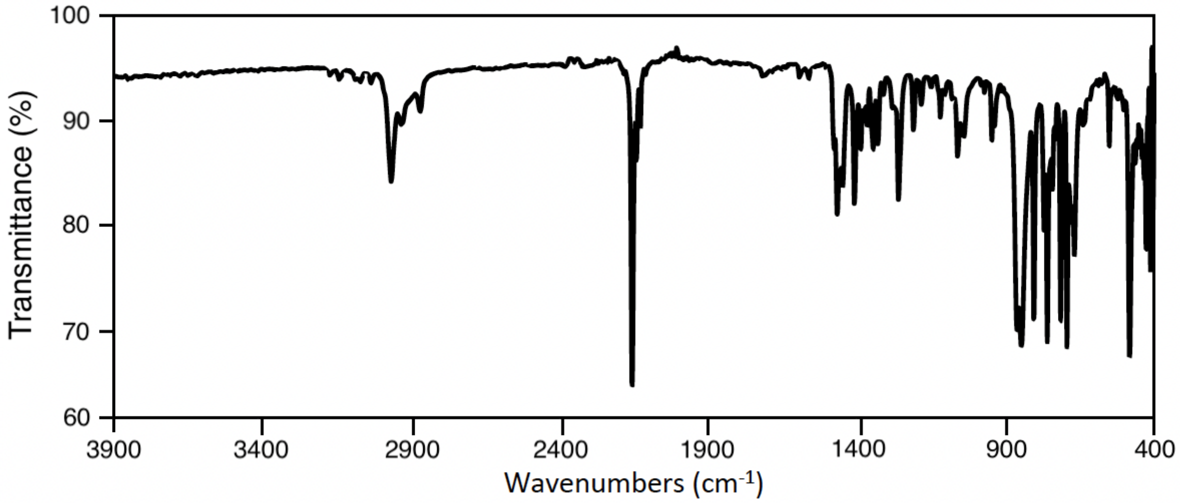

Figure S21. FT-IR spectrum of [(IDipp)Ni(CN)(μ-CN)]$_4$.

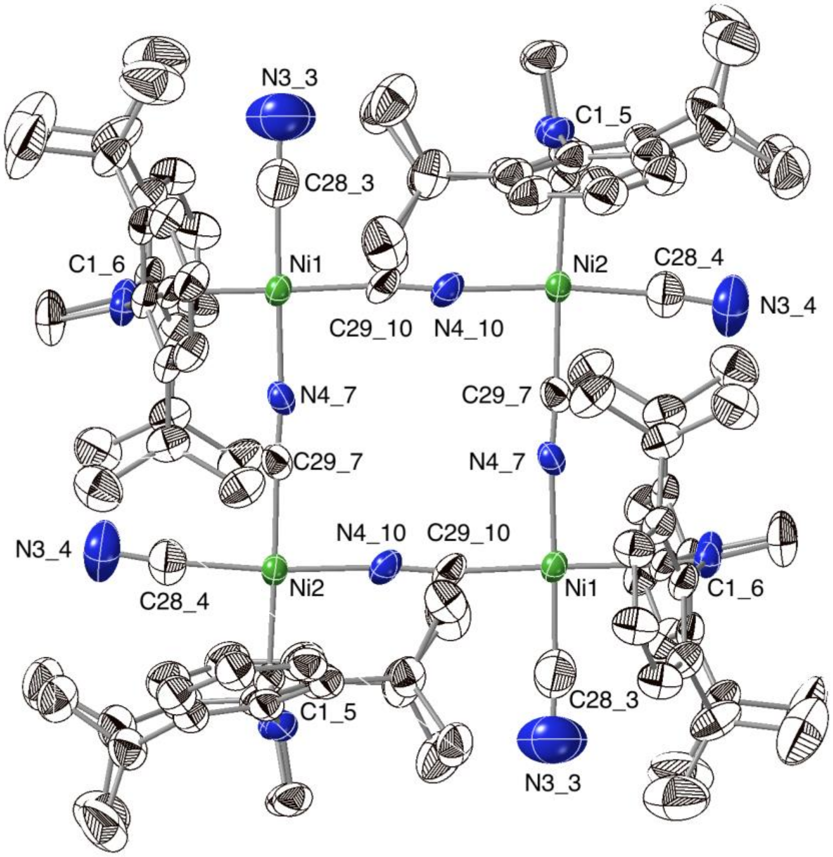

Figure S22. Solid-state structure of [(IDipp)Ni(CN)(μ-CN)]$_4$ (**3**). Second molecule in asymmetric unit (see also Figure 3 in the text), shown as 50% probability ellipsoids. Selected interatomic distances (Å) and angles (°): Ni1–C28_3, 1.882(12), C28_3–N3_3, 1.160(18), Ni1–C1_6, 1.907(6), Ni1–C29_10, 1.841(6), C29_10–N4_10, 1.123(9), Ni1–N4_7, 1.903(8); C1_6–Ni1–C28_3, 90.8(4), C28_3–Ni1–C29_10, 87.5(4), C29_10–Ni1–N4_7, 91.4(3), N4_7–Ni1_C1_6, 90.3(3), Ni1_C29_10–N4_10, 172.9(7).

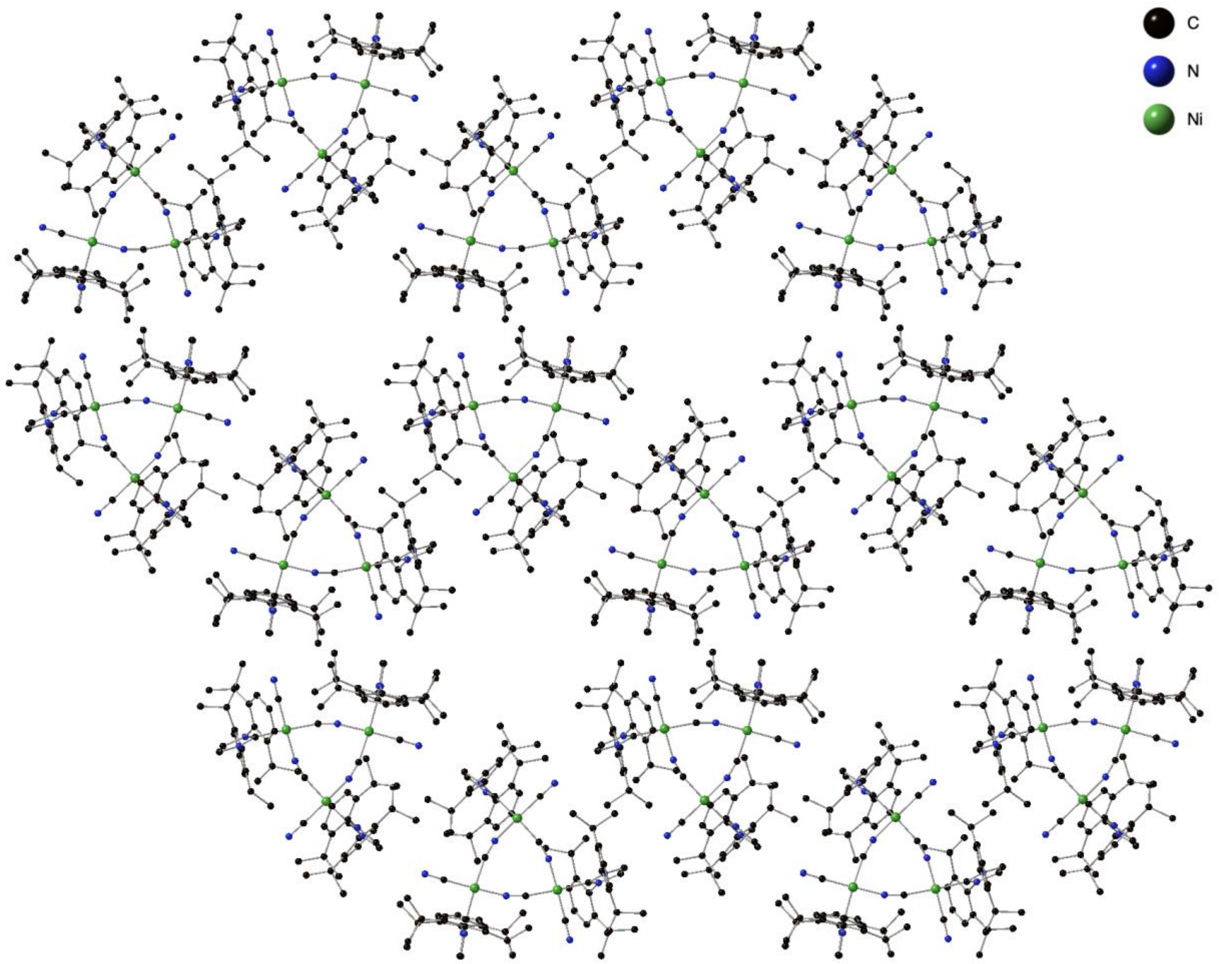

Figure S23. Solid-state structure of $[(IDipp)Ni(CN)(\mu\text{-}CN)]_3$ (**3'**), extended to show multiple unit cells. Ball-and-stick representation; hydrogen atoms and co-crystallized solvent are omitted for clarity.

References

1. Fulmer, G. R.; Miller, A. J.; Sherden, N. H.; Gottlieb, H. E.; Nudelman, A.; Stoltz, B. M.; Bercaw, J. E.; Goldberg, K. I. NMR chemical shifts of trace impurities: common laboratory solvents, organics, and gases in deuterated solvents relevant to the organometallic chemist. *Organometallics* **2010**, *29*, 2176-2179.

2. Simunic, J. L.; Pinhas, A. R. Spectroscopic identification of the nickel acylate complex. *Organometallics* **1987**, *6*, 1358-1360.

3. Lee, C. H.; Laitar, D. S.; Mueller, P.; Sadighi, J. P. Generation of a Doubly Bridging CO_2 Ligand and Deoxygenation of CO_2 by an (NHC)Ni(0) Complex. *J. Am. Chem. Soc.* **2007**, *129*, 13802-13803.
